# microRNA-155 Is Decreased During Atherosclerosis Regression and Is Increased in Urinary Extracellular Vesicles During Atherosclerosis Progression

**DOI:** 10.3389/fimmu.2020.576516

**Published:** 2020-12-17

**Authors:** Stephen Fitzsimons, Silvia Oggero, Robyn Bruen, Cathal McCarthy, Moritz J. Strowitzki, Niall G. Mahon, Nicola Ryan, Eoin P. Brennan, Mary Barry, Mauro Perretti, Orina Belton

**Affiliations:** ^1^Diabetes Complications Research Centre, School of Biomolecular and Biomedical Science, Conway Institute, University College Dublin, Dublin, Ireland; ^2^William Harvey Research Institute, Barts and the London School of Medicine, Queen Mary University of London, London, United Kingdom; ^3^Department of Pharmacology and Therapeutics, University College Cork, Cork, Ireland; ^4^School of Medicine and Medical Science, University College Dublin, Dublin, Ireland; ^5^Department of Cardiology, Mater Misericordiae University Hospital, Dublin, Ireland; ^6^Department of Vascular Surgery, St. Vincent’s University Hospital, Dublin, Ireland

**Keywords:** microRNA, miR-155, inflammation, regression of atherosclerosis, progression of atherosclerosis, macrophage, extracellular vesicles, urine

## Abstract

**Background:**

Atherosclerosis is a chronic inflammatory disease driven by macrophage accumulation in medium and large sized arteries. Macrophage polarization and inflammation are governed by microRNAs (miR) that regulate the expression of inflammatory proteins and cholesterol trafficking. Previous transcriptomic analysis led us to hypothesize that miR-155-5p (miR-155) is regulated by conjugated linoleic acid (CLA), a pro-resolving mediator which induces regression of atherosclerosis *in vivo*. In parallel, as extracellular vesicles (EVs) and their miR content have potential as biomarkers, we investigated alterations in urinary-derived EVs (uEVs) during the progression of human coronary artery disease (CAD).

**Methods:**

miR-155 expression was quantified in aortae from ApoE^−/−^ mice fed a 1% cholesterol diet supplemented with CLA blend (80:20, *cis*-9,*trans*-11:*trans*-10,*cis*-12 respectively) which had been previously been shown to induce atherosclerosis regression. In parallel, human polarized THP-1 macrophages were used to investigate the effects of CLA blend on miR-155 expression. A miR-155 mimic was used to investigate its inflammatory effects on macrophages and on *ex vivo* human carotid endarterectomy (CEA) plaque specimens (n = 5). Surface marker expression and miR content were analyzed in urinary extracellular vesicles (uEVs) obtained from patients diagnosed with unstable (n = 12) and stable (n = 12) CAD.

**Results:**

Here, we report that the 1% cholesterol diet increased miR-155 expression while CLA blend supplementation decreased miR-155 expression in the aorta during atherosclerosis regression *in vivo*. CLA blend also decreased miR-155 expression *in vitro* in human THP-1 polarized macrophages. Furthermore, in THP-1 macrophages, miR-155 mimic decreased the anti-inflammatory signaling proteins, BCL-6 and phosphorylated-STAT-3. In addition, miR-155 mimic downregulated BCL-6 in CEA plaque specimens. uEVs from patients with unstable CAD had increased expression of miR-155 in comparison to patients with stable CAD. While the overall concentration of uEVs was decreased in patients with unstable CAD, levels of CD45+ uEVs were increased. Additionally, patients with unstable CAD had increased CD11b+ uEVs and decreased CD16+ uEVs.

**Conclusion:**

miR-155 suppresses anti-inflammatory signaling in macrophages, is decreased during regression of atherosclerosis *in vivo* and is increased in uEVs from patients with unstable CAD suggesting miR-155 has potential as a prognostic indicator and a therapeutic target.

## Introduction

Atherosclerosis, characterized by the accumulation of lipid-rich, fibrous, necrotic plaques within the arterial wall is the predominant cause of heart attack and stroke (1). Atherosclerosis is classified as a chronic progressive inflammatory disease where monocyte and macrophage cells play a pivotal role. Although the cellular and molecular mechanisms and mediators in the pathogenesis of atherosclerosis have been extensively elucidated, there are a lack of studies exploring the microRNAs regulated during regression of atherosclerosis and their potential as therapeutic targets and biomarkers.

Conjugated linoleic acid (CLA) is a generic term used to describe a group of linoleic acid isomers that differ in the geometry and position of their conjugated double bonds (2). The two most abundant isomers are cis-9, trans-11(*c*-9,*t*-11) and trans-10, cis-12 (*t*-10,*c*-12). We have previously shown that dietary administration of an isomeric blend of these isomers; *c*-9,*t*-11: *t*-10,*c*-12, in an 80:20 ratio, halts progression and induces regression of pre-established atherosclerosis *in vivo via* alteration of monocyte/macrophage function (3, 4). Furthermore, the atheroprotective effects of CLA blend were mediated by increased interleukin (IL)-10 and phosphorylated-signal transducer and activator of transcription 3 (p-STAT-3) signaling and the priming of both bone marrow–derived macrophages (BMDMs) and human peripheral blood monocytes toward an M2-like phenotype (5, 6). CLA has also been shown to mediate its multiple physiological effects in other cells in part *via* regulation of microRNAs specifically, miR-499 (7), miR-23a (8), miR-107 (9), miR-27, and miR-143 (10).

Inflammatory signaling in macrophages is regulated by microRNAs which regulate gene expression through binding to complementary sequences on mRNA. Approximately 2,300 human mature microRNAs have been identified (11) which can regulate more than 30% of human genes (12). Interferon gamma (IFN-γ) and tumor necrosis factor-alpha (TNF-α) stimulated human macrophages (M1-like) have a different microRNA expression profile compared to non-stimulated (M0), IL-4 stimulated (M2a-like), and IL-10 stimulated (M2c-like) macrophages (13). Macrophage microRNAs including miR-155, miR-21 and miR-33 have been shown to play a role in macrophage polarization, the regulation of the inflammatory response, lipid uptake and cholesterol efflux (14). Among those, miR-155 is processed from the MIR155 Host Gene (*MIR155HG*), previously known as the B-cell integration cluster (*BIC*) gene (15). The role of miR-155 in the innate immune response has been extensively reviewed (16). Furthermore, we have recently described the role of miR-155 and its ability to govern inflammation in the context of atherosclerosis (17).

miR-155 is upregulated in human (18) and murine atherosclerotic lesions where its expression is localized to lesional macrophages (19). miR-155 is also upregulated in monocytes and macrophages following immune activation by pro-inflammatory stimuli such as lipopolysaccharide (LPS) and oxidized low density lipoprotein (LDL). LPS and IFN-γ–induced macrophages (M1-like) have increased miR-155 in comparison to M0 unstimulated macrophages and IL-4-induced macrophages (M2a-like) (20). Validated targets of miR-155 involved in the inflammatory response include Src homology 2 domain-containing inositol 5’-phosphatase 1 (SHIP-1) (21), the transcription factor, B-cell lymphoma protein (BCL)-6 (22) and suppressor of cytokine signaling 1 (SOCS1) (23) which result in increased reactive oxygen species (ROS), decreased efferocytosis and increased type 1 IFN signaling, respectively.

There is conflicting data as to the exact role of miR-155 in the context of atherosclerosis. In an apolipoprotein E (ApoE) knockout (^−/−^) (ApoE^−/−^) atherosclerotic mouse model with a leukocyte-specific miR-155 deficiency, plaque size and the number of lesional macrophages was decreased following partial carotid ligation (19). Similarly, ApoE^−/−^ miR-155^−/−^ double knockout mice had decreased macrophage inflammation and had reduced atherosclerotic lesion development. The regulatory effects of miR-155 in leukocyte cells were confirmed through transplantation of miR-155 deficient bone marrow into ApoE^−/−^ mice which resulted in halted atherosclerosis (24). Interestingly, injection of antagomir-155 into ApoE^−/−^ mice halted atherosclerosis development and progression (25, 26). However in contrast, there were increased atherosclerotic plaques and elevated levels of pro-inflammatory monocytes in LDL receptor (LDLR)^−/−^ mice with miR-155-deficient bone marrow (27).

Extracellular vesicles (EVs) (exosomes, microvesicles, and apoptotic bodies) contain specific patterns of microRNAs, mRNAs, and proteins (28). EVs have been characterized from a broad range of biological fluids including human plasma, saliva, breast milk (29), urine (30), peritoneal fluid and ascites (31), follicular fluid (32), and cerebral spinal fluid (33). The biomarker potential of EVs and their microRNA contents in the context of cardiovascular disease (CVD) has been reviewed extensively (34, 35). For example, patients with unstable carotid plaques had increased plasma EVs and decreased CD11b^+^ and CD45^+^ EVs (36). Patients diagnosed with a ST-elevation myocardial infarction (STEMI) had increased blood EVs and following percutaneous coronary intervention there was a decreased in intracoronary EVs (37). EV microRNAs derived from plasma also have potential as biomarkers to distinguish between different stroke subtypes (38). A set of miRNAs in urinary EVs (uEVs) from patients with Type 1 diabetes were shown to be associated with diabetic nephropathy occurrence (39). Recent work has shown that EVs from the circulation can be transferred to the urine and have potential as biomarkers for neurological diseases such as Parkinson’s disease (40). However, how reflective uEVs are of atherosclerotic plaque inflammation remains to be elucidated.

Using our validated model of atherosclerosis regression the main aim of this study was to examine the regulation of miR-155 in CLA-induced regression. In parallel, we also investigated miR-155 expression and alterations in uEVs in human coronary artery disease (CAD) progression.

## Materials and Methods

### Animals and Diets

Murine aortic samples were obtained from a previously published study performed by McCarthy et al. (5). All animal experiments were performed in accordance with institutional guidelines and ethics committees, and in compliance with international laws. In brief, ApoE^−/−^ mice on a C57BL/6 background were obtained from the Jackson Laboratory (Bar Harbor, ME, USA). The CLA blend diet consisted of an 80:20 ratio of *c*-9, *t*-11-CLA: *t*-10, *c*-12-CLA (Stepan Lipid Nutrition, Wormerveer, Holland) which was incorporated into 1% cholesterol chow (Special Dietary Services, Essex, UK). ApoE^−/−^ mice fed a 1% cholesterol chow diet (1% CD) or normal chow diet (ND) group were used as controls. ApoE^−/−^ mice were randomized at 40 days to receive either a ND for 12 weeks, a 1% CD for 12 weeks or a 1% CD for 12 weeks where the final 4 weeks were supplemented with 1% CLA blend (1% CD + CLA) to induce regression (3–5).

### Ingenuity Pathway Analysis

Previously, we performed transcriptome profiling of murine aortae comparing the 1% CD + CLA group to the 1% CD group (McCarthy 2013). Transcripts with a false discovery rate (FDR) p-value <0.05 and fold change >1.7 were deemed statistically significant. miRNA networks were determined by Upstream Regulator analysis of differentially expressed genes using Ingenuity Pathway Analysis (IPA) Z-score algorithm (Ingenuity Systems, Qiagen). A cut-off Z-score of ≥ 2 or ≤ −2, and p < 0.05 was deemed significant.

### Cell Culture, Macrophage Polarization, and Treatment

THP-1 monocytes (ATCC^®^ TIB-202™, Teddington, Middlesex, UK, CLS Cat# 300356/p804_THP-1, RRID : CVCL_0006) were differentiated to M0 macrophages and further polarized to M1 and M2 as previously published (41). In brief, THP-1 monocytes were treated with 320 nmol/L phorbol 12-myristate 13-acetate (PMA) (Sigma-Aldrich, Dorset, UK) for 72 h. Cells were rested in complete Roswell Park Memorial Institute media (RPMI 1640+GlutaMAX (Gibco, Thermo Fisher Scientific, UK) supplemented with 1% penicillin/streptomycin and 10% fetal bovine serum) for 24 h followed by a 48-h treatment with LPS (100 ng/ml) (InvivoGen, Toulouse, France) and IFN-γ (20 ng/ml) (R&D Systems, Abingdon, UK) to induce M1 polarization and IL-4 (20 ng/ml) and IL-13 (20 ng/ml) (R&D Systems, Abingdon, UK) to induce M2 polarization. M0, M1 and M2 macrophages were then treated with CLA blend (*c-*9, *t*-11-CLA: *t*-10, *c*-12-CLA in a ratio of 80:20 at 25µmol/L) (Cayman Chemicals, Michigan, USA) or vehicle control, dimethyl sulfoxide (DMSO) (Sigma-Aldrich, Dublin, Ireland) at 0.1% for 18 h in RPMI serum-free media. RNA was isolated from cells using an RNeasy Mini Kit (Qiagen Ltd, Manchester, UK) for quantitative real-time polymerase chain reaction (qRT-PCR) and protein was harvested for Western blotting using radioimmunoprecipitation assay (RIPA) buffer (Sigma-Aldrich, Dorset, UK).

### Flow Cytometry Analysis

Phenotypic changes in untreated, INF-γ/LPS- and IL-4/IL-13–treated macrophages were analyzed by flow cytometry using the Accuri™ C6 Flow Cytometer (BD Biosciences, San Jose, USA). Macrophages were washed in PBS supplemented with 0.02% bovine serum albumin and counted. One hundred thousand cells per sample were blocked using Human BD Fc Block™ (5 µg/ml) (BD Biosciences) and then double stained for 30 min at 4°C with human leukocyte antigen-DR isotype (HLA-DR) FITC conjugated (diluted 1:100) and mannose receptor (MR) APC conjugated (diluted 1:100) as outlined in **Supplemental Methods Table 1**. Single stained antibodies, consisting of a single antibody mixed with macrophages, were used as the fluorescence minus one (FMO) controls. For gating strategies, unstained untreated macrophages were used as the control (**Supplemental Figure 1**). Ten thousand gated events were recorded per sample. Fluorescence signal was detected using the 3 blue, 1 red laser configuration and the FL-1 (filter wavelength: 533/30; FITC signal) and FL-4 (filter wavelength: 675/25; APC signal) channel of the Accuri^™^. Median fluorescence intensity values and the percentages of positive cells were determined for each sample and analyzed using FCS Express 6 (De Novo Software, Pasadena, USA).

### Transfection of THP-1 M0 Macrophages With miR-155 Mimic

THP-1 monocytes were seeded at 5 × 10^5^ cells/ml in complete RPMI media and treated with PMA (320 nmol/L) for 72 h. Cells were rested in complete RPMI media for 24 h followed by treatment with human hsa-miR-155-5p miRVana^®^ miRNA mimic (20 nmol/L) (Ambion, Thermo Fisher Scientific, UK) for 24 h using Lipofectamine 3000™ (Invitrogen, Thermo Fisher Scientific, UK), Opti-MEM^®^ I Media (Gibco, Thermo Fisher Scientific, UK) and complete RPMI media. Lipofectamine-treated cells (Untreated) and miRVana^®^ microRNA mimic Negative Control (Ambion, Thermo Fisher Scientific, UK) were used as the controls. Supernatants were removed and cells were stimulated with LPS (100 ng/ml) for 18 h. Total RNA was isolated using TRIzol™ Reagent (Ambion, Thermo Fisher Scientific, UK).

### Enzyme-Linked Immunosorbent Assay

THP-1 supernatants were analyzed by enzyme-linked immunosorbent assay (ELISA) for human IL-10, IL-1β, and TNF-α (Thermo Fisher Scientific, UK) according to the manufacturer’s instructions.

### Western Blotting

Protein was quantified using a Bradford Assay (Bio-Rad, Dublin, Ireland) according to the manufacturer’s instructions. Proteins were separated by sodium dodecyl sulfate-polyacrylamide gel electrophoresis (SDS-PAGE) on either 10% or 12% resolving gels. Gels were transferred to a nitrocellulose membrane (GE Healthcare Amersham™ Protran™, Life Sciences, Thermo Fisher Scientific, Ireland). Membranes were blocked and incubated with diluted primary antibody overnight at 4°C. Details of primary antibodies are outlined in **Supplemental Methods Table 2**. Either β-actin or glyceraldehyde 3-phosphate dehydrogenase (GAPDH) were used as loading/housekeeping controls for densitometry analysis which was performed using ImageJ Software (National Institutes of Health, US). All targets of interest were normalized to the control treatment or control group and then normalized to the loading/housekeeping control and represented as protein expression relative to control.

### qRT-PCR

RNA was extracted from cells, aortae, EVs and plaque using TRIzol™ Reagent (Ambion, Thermo Fisher Scientific, UK) as per manufacturer’s instructions. The NanoDrop™ 2000 (Life Technologies Ltd., Paisley, UK) was used to determine RNA quality and quantity. For microRNA analysis, 100 ng of total RNA was reverse transcribed to microRNA cDNA specific for the respective Taqman hydrolysis probes using the Applied Biosystems TaqMan microRNA reverse transcription kit (Biosciences, Dublin, Ireland). For mRNA analysis, 500 ng of total RNA was reverse transcribed. TaqMan hydrolysis probes are listed in **Supplemental Methods Table 3**. Analysis was performed using the Applied Biosystems QuantStudio™ 7 Flex Real-Time PCR System.

### Study Population

Full ethical approval was obtained from the Ethics Committee at The Mater Misericordiae University Hospital, Dublin, Ireland for the collection of urine samples from patients with CAD. The study was carried out in accordance with the World Medical Association’s Declaration of Helsinki. All participants gave informed written consent. The clinical characteristics of the patients are outlined in **Table 1**. Patients that presented with Acute Coronary Syndrome (STEMI, non-STEMI or unstable angina) were grouped under the term “Unstable CAD” (n = 12). Patients that presented with stable CAD were grouped under the term “Stable CAD” (n = 12).

**Table 1:** Demographics from The Mater Misericordiae University Hospital from patients diagnosed with stable and unstable CAD.

Demographics	Flow cytometry			Nanoparticle tracking analysis		
CAD Status	Stable (n = 12)	Unstable (n = 12)	p-value	Stable (n = 13)	Unstable(n = 12)	p-value
**Diagnosis, n (%)**						
*-STEMI*	0	3 (25%)		0	2 (16%)	
*-NSTEMI*	0	9 (75%)		0	5 (42%)	
*-Unstable Angina*	0	0		0	5 (42%)	
*-Stable*	12 (100%)	0		13 (100%)	0	
**Age (Mean ± SD)**	69.5 ± 9.3	68.3 ± 10.2	0.75 (ns)	71.6 ± 7.8	64.6 ± 12.3	0.14 (ns)
**Gender, n (%)**						
*-Male*	9 (75%)	9 (75%)	1 (ns)	7 (54%)	7 (58%)	1 (ns)
*-Female*	3 (25%)	3 (25%)		6 (46%)	5 (42%)	
**Diabetic Status, n (%)**						
*-Type 1*	1 (8%)	0		0	0	
*-Type 2*	4 (33%)	6 (50%)		0	1 (8%)	
*-No*	5 (58%)	6 (50%)		13 (100%)	11 (92%)	
**Medication, n (%)**						
-*Statin*	12 (100%)	7 (58%)		12 (92%)	9 (75%)	
**Creatinine, mean ± SD (µmol/L)**	94.8 ± 23.2	96.83 ± 29.36	0.75 (ns)	80.5 ± 21.8	80.7 ± 16.1	0.64 (ns)
**Prior Revascularization, n (%)**						
*-Yes*	3 (25%)	8 (67%)	0.09 (ns)	6 (46%)	3 (25%)	0.4 (ns)
*-No*	9 (75%)	4 (33%)		7 (54%)	9 (75%)	
**Premature CAD, n (%)**						
*-Yes*	3 (25%)	2 (16%)	1 (ns)	0	0	
*-No*	9 (75%)	10 (84%)		13 (100%)	12 (100%)	
**Hypertension**						
*-Yes*	8 (67%)	7 (58%)	1 (ns)	9 (69%)	6 (50%)	0.4 (ns)
*-No*	4 (33%)	5 (42%)		4 (31%)	6 (50%)	

Forty-nine samples selected for uEV analysis were grouped into stable and unstable depending on their diagnosis. Patient demographics include diagnosis; stable versus unstable (STEMI, non-STEMI, unstable angina), age, gender, diabetic status, medication, creatinine levels, prior revascularization, premature CAD, and hypertension. Statistical analysis was performed using Fisher’s exact test to analyze all categorical variables and a non-parametric Mann Whitney U test to analyze age and creatinine concentration (ns, not significant).

### Urinary Extracellular Vesicle Isolation and Characterization

EVs were isolated from urine *via* serial benchtop centrifugation (Eppendorf Centrifuge 5417R) at 4°C. In brief, samples were centrifuged in 1.5 ml Eppendorfs at 2,000x*g* for 20 min, the supernatant was then centrifuged at 17,000x*g* for 45 min. For nanoparticle tracking analysis (NTA) and qRT-PCR, the supernatant was then centrifuged at 24,000x*g* for 60 min, the EV pellet was washed in double filtered sterile PBS and then the centrifugation step was repeated and the EVs were resuspended in PBS or TRIzol™. For flow cytometry and transmission electron microscopy (TEM), the supernatant was centrifuged at 20,000x*g* for 90 min, the EV pellet was washed in PBS and then the centrifugation step was repeated and the EVs were resuspended in PBS. For both NTA and flow cytometry analysis, four EV pellets were pooled and resuspended in 300 µl of PBS. For TEM four EV pellets were pooled and resuspended in 50 µl of PBS. For qRT-PCR analysis, four pellets were pooled in 500 µl of TRIzol™ reagent.

### Transmission Electron Microscopy of uEVs

EV sample derived from 6 ml of urine (four EV pellets) was resuspended in 50µL double filtered sterile PBS. Formvar carbon coated copper grids (G200-Cu) (EMS, Pennsylvania, USA) were placed on top of 10 µl of EV sample. Grids were then washed in PBS and fixed using 2.5% of glutardialdehyde (Merck, Darmstadt, Germany) for 10 min. Grids were washed in dH_2_O and incubated with 2% uranyl acetate (Agar Scientific, Essex, UK) for 15 min followed by two rinses and a 10-min incubation on ice in a solution of 1.8% methyl cellulose (Sigma-Aldrich, Dorset, UK) and 0.4% uranyl acetate in dH_2_O. Excess liquid was removed, and samples were air-dried for 20 min. Images were taken using an FEI Tecnai 12 Transmission Electron Microscope with the acceleration voltage set at 120 kv.

### Nanoparticle Tracking Analysis of uEVs

The size distribution and concentration of EVs resuspended in double filtered PBS was measured by NTA using the NanoSight NS300 (Malvern Panalytical Ltd, Worcester, UK). (**Supplemental Figure 2**). EV samples were diluted 1:10 in 1-ml PBS and analyzed under constant flow conditions; flow rate 50, temperature 25°C. Fifteen 60-s videos were recorded for each sample using the appropriate camera level and focus. Videos were analyzed using the NanoSight NTA 3.1 software (Malvern Panalytical Ltd, UK) using 10 nm sized bins and a set detection threshold, while the concentration was calculated accounting for the dilution factor. Data from each sample were expressed as the mean ± standard error of the mean (SEM) of the 15 recordings. Graphs were generated using RStudio (Boston, MA, USA) and GraphPad Prism 5.0c (GraphPad Software Inc, San Diego, CA, USA).

### Flow Cytometry of uEVs

EVs in 30 μl of PBS were labelled with either 50 μmol/L of Bodipy Maleimide Fluorescein or Bodipy Texas Red (Life Technologies, Carlsbad, USA) and diluted in PBS. Samples were centrifuged at 20,000x*g* for 90 min at 4°C and then EV pellets were labelled with the respective antibodies listed in **Supplemental Methods Table 1** which were diluted 1:30 in PBS (total volume 30 μl). Analysis of EVs was performed using an ImageStream^®^ X Mark II Flow Cytometer (Amnis^®^, Seattle, USA) set to a slow flow rate and 60X magnification. Following bead compensation, the “Remove Beads” setting was turned on after loading each sample. Sample acquisition was initiated once the flow rate had stabilized. A gate was created to store all events, followed by setting a stopping gate set to capture the EV population which is characterized by low side scatter and medium to low fluorescence of the Bodipy label. FMO controls were used for gating on all protein antigen-positive events. CD63, CD14, CD16, CD45, and CD11b labelled EV events were acquired (**Supplemental Figure 3**). Sample graphs of the gating strategy are shown in **Supplemental Figure 4**. Images of single uEVs are shown in **Supplemental Figure 5**. A minimum of 10,000 EV objects per sample were analyzed in the particles gate. Flow cytometry analysis was performed using the IDEAS 6 Software (Amnis^®^, Seattle, USA).

### Statistical Analysis

Results generated were analyzed using GraphPad Prism 5.0c (GraphPad Software Inc., San Diego, CA, USA). Data are presented as mean ± SEM which is represented on each graph using an error bar. THP-1 data were analyzed using individual paired t-tests comparing all columns of interest. ApoE^−/−^ *in vivo* experiments, *ex vivo* plaque transfections and human uEV experiments were analyzed using unpaired nonparametric tests (Mann-Whitney U test) to compare all columns of interest. Patient demographics were analyzed using a Fisher’s exact test to analyze all categorical variables and a nonparametric Mann-Whitney U test for age and creatinine. Statistical significance was considered when *p < 0.05, **p < 0.01, and ***p < 0.001.

## Results

### Dietary CLA Reduces Aortic miR-155 Expression in ApoE^−/−^ Fed a 1% Cholesterol Diet

**Figure 1:**
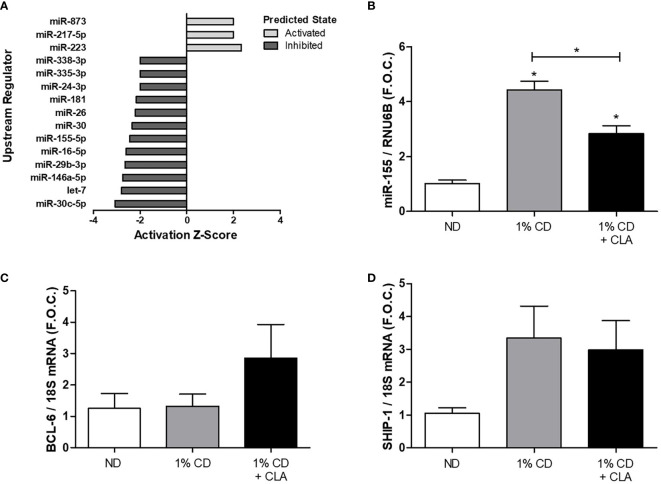
Dietary CLA blend decreased aortic miR-155 expression in ApoE^−/−^ mice. ApoE^−/−^ mice were fed a ND or a 1% CD for 12 weeks or a 1% CD for 12 weeks with the final 4 weeks supplemented with 1% CLA blend (1% CD + CLA). Aortae were dissected and RNA was isolated. Previously, transcriptomic analysis was performed on murine aortae from the 1% CD + CLA group and the 1% CD group by McCarthy et al. **(A)** *In silico*, IPA analysis was performed to identify upstream regulators of the CLA-responsive gene set (n = 1082; FDR p < 0.05; Fold-change >1.7). The activation Z-score (≥ 2 or ≤ −2) and upstream miRNA regulators identified were graphed, the overlap p-value was set to p < 0.05. **(B)** miR-155 expression was analyzed by qRT-PCR using RNU6B as the reference gene. **(C)** BCL-6 and **(D)** SHIP-1 expression analyzed by qRT-PCR using 18S as the reference gene and graphed as fold over control (F.O.C.). qRT-PCR results are expressed as mean ± SEM (4 ≤ n ≤ 9). Statistical analysis was performed using a Mann-Whitney U test. *p < 0.05 was considered statistically significant. * over the columns are comparisons with the ND, control group. Significance between other groups is indicated by capped lines.

To elucidate the effects of CLA blend on miRNA regulators in a murine model of atherosclerosis we used a model from our previous studies, which demonstrated that CLA blend induced regression of pre-established atherosclerosis. ApoE^−/−^ mice fed a 1% CD for 8 weeks, and maintained on this diet for a further 8 weeks with 1% CLA supplementation showed reduced aorta lesion size by approximately 30% when compared to controls (3). The model has been extensively validated, most recently by Bruen et al. (4). In a separate 12-week study using this model, we previously performed transcriptomic analysis on aortae from ApoE^−/−^ mice fed either a 1% CD or a 1% CD supplemented with 1% CLA blend for the final 4 weeks. Here we defined the aortic transcriptome responses to CLA and demonstrated that CLA supplemented mice had increased aortic IL-10 signaling (McCarthy, 2013). In order to further investigate putative miRNA regulatory networks in response to CLA treatment, we performed an *in silico* analysis of these transcriptomics data. Here, we performed an upstream regulator analysis of the CLA-responsive gene set (n = 1082; FDR p < 0.05; Fold-change >1.7) and identified 15 miRNA networks predicted to be modulated in response to CLA (**Figure 1A**). Interestingly, 12/15 miRNA networks are predicted to be inhibited by CLA supplementation. Among these, miR-155 is predicted to be inhibited by CLA (Z-score −2.447; p = 0.015), with multiple genes known to be regulated by miR-155 predicted to be upregulated following CLA-supplementation (**Supplemental Figure 6**). Previously, miRNA-155 deficiency was demonstrated to decrease macrophage inflammation and attenuate atherogenesis in ApoE^−/−^ mice (24). miR-155 was reported to promote atherosclerosis by inhibition of BCL-6 in macrophages (19) further highlighting that miR-155 plays a key regulatory role in atherosclerotic development which warrants further investigation. Thus, to understand miR-155 regulation in the context of regression of atherosclerosis, we investigated the effect of CLA on miR-155 regulation in aortic tissue.

miR-155 expression was significantly increased in aortic tissue from ApoE^−/−^ mice fed a 1% CD compared to the ND group (ND 1.02 ± 0.12 vs. 1%CD 4.44 ± 0.30 fold change, *p < 0.05) (**Figure 1B**). This was coincident with an increase in aortic macrophages as determined by increased protein expression of pan macrophage marker, CD68, and vascular cell adhesion molecule-1 (VCAM-1) (**Supplemental Figure 7**). Notably, miR-155 expression was significantly decreased in aortae from mice fed a 1% CD with 4 week CLA supplementation compared to that from 1% cholesterol controls (1%CD 4.44 ± 0.30 vs. 1%CD ± CLA 2.84 ± 0.28 fold change, *p < 0.05), (**Figure 1B**). This was associated with a trend toward an increase in aortic BCL-6 mRNA expression, in the CLA supplemented group (**Figure 1C**), although this was not significant. Aortic SHIP-1 mRNA expression was increased in the 1% CD group when compared to ND group, however this was not significant and was not altered by CLA treatment (**Figure 1D**). The data shows that miR-155 expression is increased during the development of atherosclerosis and that CLA decreases aortic miR-155 expression during regression of atherosclerosis.

### Effect of CLA on miR-155 in Polarized THP-1 Macrophages

**Figure 2:**
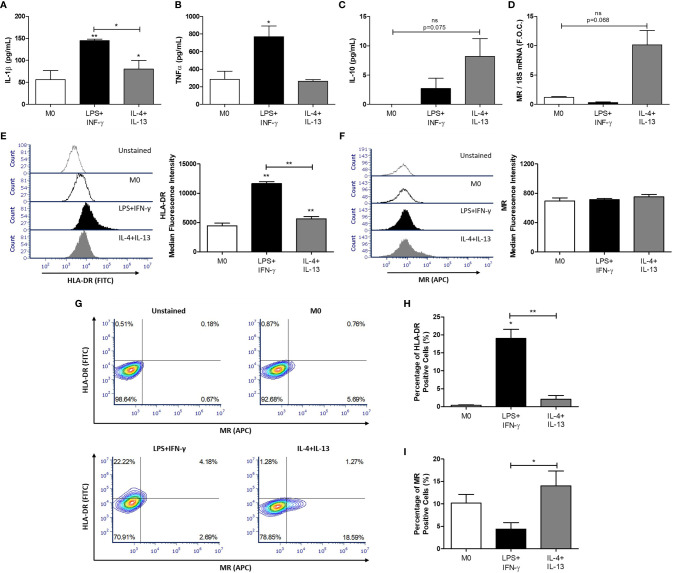
Polarization of THP-1 macrophages. THP-1 monocytes were polarized into THP-1 macrophages (M0) using PMA (320 nmol/L) for 72 h, after which cells underwent a 24 h rest phase to allow the M0 phenotype to develop. Macrophages were then treated for 48 h with 100 ng/ml LPS and 20 ng/ml IFN-γ to polarize cells to M1 macrophages and 20 ng/ml IL-4 and IL-13 for M2 macrophages. The supernatants were analyzed by ELISA for **(A)** IL-1β, **(B)** TNF-α, and **(C)** IL-10 secretion. RNA was analyzed for **(D)** MR expression by qRT-PCR using 18S rRNA (18S) as the reference gene and graphed as fold over control (F.O.C.). Flow cytometry analysis was used to determine the median fluorescence intensity of **(E)** HLA-DR (FITC) and **(F)** MR (APC) and illustrated using a representative histogram plot. **(G)** Quadrant regions were used to detect the percentage of macrophages positive for HLA-DR (FITC) and MR (APC) following the respective treatments. The percentage of **(H)** HLA-DR and **(I)** MR positive cells was determined and graphed. Error bars are representative of independent experiments performed in duplicate for TNF-α (n = 3), IL-10 (n = 4), IL-1β (n = 7), MR (n = 3) and HLA-DR (n = 3). Statistical analysis was carried out performing multiple paired t-tests comparing all groups. *p < 0.05 and **p < 0.01, were considered statistically significant. * over the columns are comparisons with the control M0 group. Significance between other groups is indicated by capped lines. ns, not significant.

We have previously shown that CLA mediates its atheroprotective effect in part by inducing a M2-like pro-resolving macrophage phenotype *in vitro* and *in vivo* (4–6). To investigate the effect of CLA on miR-155 in polarized macrophages, human THP-1 monocytes were polarized to establish a model of M0-, M1-, and M2-like macrophages. IL-1β cytokine secretion was significantly increased in the supernatants from LPS and INF-γ–treated macrophages (M1-like) when compared to untreated controls (M0) (M0 56.45 ± 20.33 pg/ml vs. LPS/INF-γ–treated 144.9 ± 3.69 pg/ml, **p < 0.01) (**Figure 2A**). Similarly, TNF-α secretion was increased in LPS and INF-γ–treated macrophages (M0 284.9 ± 93.79 pg/ml vs. LPS/INF-γ–treated 770.5 ± 121.7 pg/ml, *p < 0.05) (**Figure 2B**). IL-10 secretion was increased from IL-13 and IL-4 treated macrophages (M2-like) (M0 0 ± 0 pg/ml vs. IL-4/IL-13–treated 8.18 ± 3.05 pg/ml, p = 0.07) (**Figure 2C**). qRT-PCR analysis showed an increase in expression of the M2 macrophage marker, MR, in IL-4– and IL-13–treated macrophages (M0 1.17 ± 0.13 vs. IL-4/IL-13–treated 10.13 ± 2.24, fold change, p = 0.06) (**Figure 2D**). Flow cytometry analysis was performed to further validate the efficiency of M1 and M2 macrophage polarization. The median fluorescence intensity of cells positive for the major histocompatibility complex class II molecule, HLA-DR, previously identified as an M1-like macrophage marker (42, 43), were significantly upregulated in LPS and IFN-γ treated macrophages when compared to M0 and IL-4- and IL-13-treated macrophages (**Figure 2E**). The median fluorescence intensity of MR, previously used as an M2-like macrophage marker (42, 44, 45), was not increased (**Figure 2F**). However, the percentage of cells positive for HLA-DR and MR, was also analysed (**Figure 2G**) and HLA-DR was significantly upregulated in LPS and IFN-γ treated macrophages (**Figure 2H**) and MR was significantly upregulated in IL-4- and IL-13-treated macrophages (**Figure 2I**).

**Figure 3:**
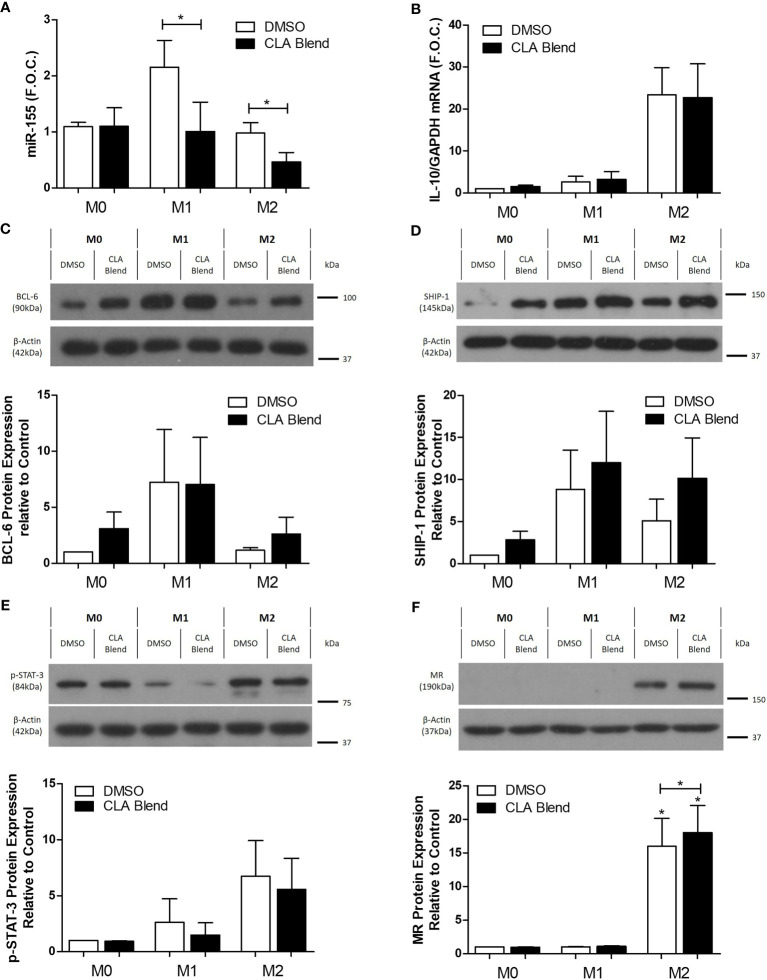
CLA decreased miR-155 and increased protein expression of miR-155 targets, BCL-6 and SHIP-1, in polarized M2 THP-1 macrophages. Polarized THP-1 macrophages were treated with CLA blend (25 µmol/L) for 18 h using DMSO (0.1%) as the control. microRNA was converted to cDNA which then underwent qRT-PCR analysis for **(A)** miR-155 expression using RNU48 and RNU6B as the reference genes. Error bars are representative of four independent experiments (n = 4) carried out in duplicate and averaged and graphed as fold over control (F.O.C.). mRNA was analyzed by qRT-PCR for **(B)** IL-10 expression (n = 5). Western blotting and densitometry analysis were performed on **(C)** BCL-6 (n = 4), **(D)** SHIP-1 (n = 5), **(E)** p-STAT-3 (n = 4), and **(F)** MR (n = 4) using β-Actin and GAPDH as controls. Statistical analysis was carried out using multiple paired t-tests where *p < 0.05 represent statistical significance. * over the columns represents statistical significance when compared to the control group M0 treated with DMSO. Significance between other groups is indicated by capped lines.

Polarized THP-1 macrophages were treated with 25 µmol/L CLA blend for 18 h using 0.1% DMSO as a control. CLA blend decreased the expression of miR-155 compared to DMSO in both M1 (DMSO 2.1 ± 0.48 vs. CLA 1.0 ± 0.52 fold change, *p < 0.05) and M2 macrophages (DMSO 0.97 ± 0.18 vs. CLA 0.46 ± 0.16 fold change, *p < 0.05) (**Figure 3A**). Interestingly, IL-10 expression, previously shown to suppress miR-155 expression, was unchanged by CLA treatment (**Figure 3B**). p-Akt, previously shown to suppress miR-155 expression, was increased by CLA, however this occurred only in M1-like macrophages (**Supplemental Figure 8**). The mRNA expression of the miR-155 targets, BCL-6 and SHIP-1, was unchanged following CLA treatment (**Supplemental Figure 9**). However, at the protein level, CLA blend increased BCL-6 and SHIP-1 in M2-like macrophages (**Figures 3C, D**) and SHIP-1 protein was increased by CLA in all macrophage phenotypes (**Figure 3D**) although these results were not statistically significant. p-STAT-3 expression was increased in M2-like macrophages when compared to M1-like macrophages, however, it was unaffected by CLA blend (**Figure 3E**). Interestingly, while MR protein expression was increased in the M2 macrophages (M0-DMSO 1 ± 0.00 vs. M2-DMSO 16 ± 4.14 fold change, *p < 0.05), there was a further small but significant increase in MR protein expression following treatment with CLA blend (M2-DMSO 16 ± 4.14 vs. M2-CLA 18.03 ± 4.04 fold change, *p < 0.05) suggesting it promotes an M2-like phenotype (**Figure 3F**).

### miR-155 Mimic Decreases the Anti-Inflammatory Proteins, BCL-6, and p-STAT-3 in Macrophages

**Figure 4:**
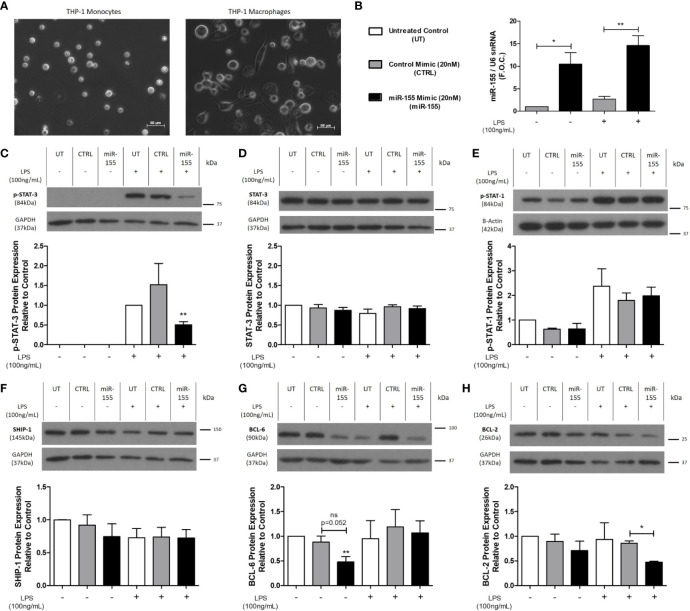
p-STAT-3, BCL-6, and BCL-2 protein expression is decreased following miR-155 mimic transfection of THP-1 M0 macrophages. **(A)** Light microscopy images of THP-1 monocytes which were differentiated to macrophages using PMA (320 nmol/L) for 72 h followed by a 24-h rest phase. Macrophages were transfected for 24 h with 20nmol/L of miR-155 mimic using Lipofectamine™ 3000. Scrambled negative control mimic and Lipofectamine™ 3000 only (untreated) were used as controls. The media was removed, and cells were stimulated with LPS (100 ng/ml) for 18 h using dH_2_O as the control. Cells were harvested for RNA and protein. Transfection efficacy was analyzed for **(B)** miR-155 expression (n = 5) by qRT-PCR and graphed as fold over control (F.O.C.). Western blotting and densitometry analysis were performed on **(C)** p-STAT-3 (n = 4), **(D)** Total STAT-3 (n = 3), **(E)** p-STAT-1 (n = 3), **(F)** SHIP-1 (n = 5), **(G)** BCL-6 (n = 5), and **(H)** BCL-2 (n = 3) expression, using β-actin and GAPDH as the loading controls. Statistical analysis was performed using multiple paired t-tests where *p < 0.05 and **p < 0.01 represented statistical significance. * over the columns represent statistical significance when compared to the untreated control group. Significance between other groups is indicated by capped lines.

THP-1 monocytes were differentiated to macrophages using 320nmol/L PMA (**Figure 4A**) prior to transfection with the miR-155 mimic to elucidate the inflammatory effects of miR-155. M0 macrophages were transfected with the miR-155 mimic for 24 h followed by stimulation with LPS for 18 h. qRT-PCR confirmed successful transfection of the miR-155 mimic both in the absence (mimic control 2.71 ± 0.59 vs. miR-155 mimic 14.59 ± 2.17 fold change, *p < 0.05) and presence of LPS (mimic control 1.02 ± 0.01 vs. 10.48 ± 2.56 fold change, **p < 0.01) (**Figure 4B**). Phosphorylation of the anti-inflammatory signaling protein, STAT-3, was significantly decreased by the miR-155 mimic in the presence of LPS (untreated control 1 ± 0.00 vs. miR-155 mimic 0.5 ± 0.07, *p < 0.05) while there was no change in total STAT-3 (**Figures 4C, D**). Interestingly, p-STAT-1 and SHIP-1 were unchanged by transfection of the miR-155 mimic (**Figures 4E, F**). However, the miR-155 mimic suppressed BCL-6 in comparison to the untreated control (untreated control 1 ± 0.00 vs. miR-155 mimic 0.47 ± 0.11, *p < 0.05) (**Figure 4G**). Furthermore, the anti-apoptotic protein, BCL-2, was also significantly decreased in comparison to the control mimic treated with LPS (control mimic ± LPS 0.85 ± 0.04 vs. miR-155 mimic ± LPS 0.47 ± 0.01 fold change, *p < 0.05) (**Figure 4H**). In addition, the effect of miR-155 mimic and LPS in human *ex vivo* carotid plaque tissue was investigated (**Supplemental Figure 10**). Previously, miR-155 and *MIR155HG* has been shown to be upregulated in atherosclerotic plaques compared to normal veins and arteries respectively (46, 47). Thus, to further investigate the role of miR-155 upregulation an *ex vivo* model of atherosclerosis was employed as described in **Supplemental Methods 1**. We have recently utilized this *ex vivo* plaque model to investigate the therapeutic potential of let-7 miRNA in diabetes-associated atherosclerosis (48). Following transfection of miR-155 mimic into plaque specimens, SHIP-1 was unchanged and although there was a trend toward decreased expression of BCL-6, this was not statistically significant. However, both SHIP-1 and BCL-6 were significantly changed by stimulation with LPS highlighting their potential role in the inflammatory response (**Supplemental Figure 10**).

### uEV Concentration Is Altered During CAD Progression

**Figure 5:**
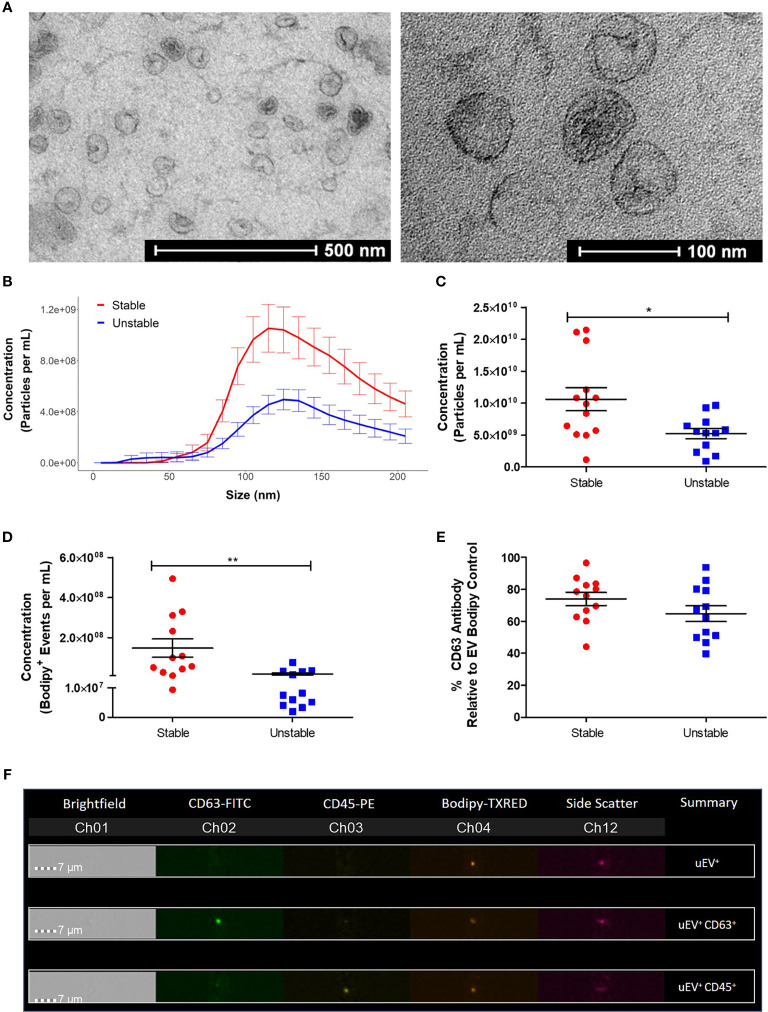
Patients with unstable CAD have a decreased concentration of uEVs. EVs were isolated from 6 ml of urine using a benchtop centrifugation protocol. **(A)** Images of uEVs were taken using an FEI Tecnai 12 Transmission Electron Microscope (Magnification: 43,000× and 135,000×). **(B)** The total concentration of particles (5-200nm) was determined by NTA using the NanoSight NS300. **(C)** R Studio analysis was performed to compare the size distribution and concentration of the NTA data. **(D)** The EV (Bodipy^+^) concentration was determined by flow cytometry using the ImageStream™X Mark II. **(E)** The percentage of CD63^+^ Bodipy^+^ EVs was also determined. **(F)** Images of EVs stained with Bodipy and positive for the fluorescent antibodies CD63 and CD45 were obtained using the ImageStream™X Flow Cytometer. Statistical analysis was performed using nonparametric Mann Whitney U tests where *p < 0.05 and **p < 0.01 represent statistical significance. Statistical significance between groups is indicated by capped lines. ns, not significant.

Urine samples were obtained from patients with stable and unstable CAD attending The Mater Misericordiae University Hospital. Patient details are described in **Table 1**. uEV from CAD patients were robustly characterized by TEM, NTA and flow cytometry, which were used to identify any alterations in the uEV profile during CAD progression. TEM analysis of human uEVs demonstrated that round cup shaped vesicles approximately 50–100 nm in size were present following isolation and fixation (**Figure 5A**). NTA data further confirmed that particles isolated were within the small EV size range with the majority between 100 and 150 nm in size (**Figure 5B**). Importantly, NTA also showed that the average concentration of uEVs in unstable CAD patients was significantly decreased when compared to stable CAD patients (unstable 5.6 × 10^9^ ± 1.0 × 10^9^ particles/ml vs. stable 1.2 × 10^10^ ± 2.0 × 10^9^ particles/ml, *p < 0.05) (**Figure 5C**). There was no significant difference between the mean and the mode particle size between stable and unstable CAD patients, however there was a significant linear correlation between creatinine concentration and particle concentration in patients with unstable CAD (**Supplemental Figure 11**).

Flow cytometry analysis was performed on the EV samples, staining membranes with Bodipy to detect the EV population, characterized by low side scatter and medium to low fluorescence intensity. Similar to the NTA data, these analyses revealed a significant decrease in the concentration of Bodipy^+^ uEVs from unstable CAD patients compared to stable CAD patients (stable 1.47 × 10^8^ ± 4.5 × 10^7^ Bodipy^+^ uEVs/ml vs. unstable 1.88 × 10^7^ ± 6.3 × 10^6^ Bodipy^+^ uEVs/ml, **p < 0.01) (**Figure 5D**). The association between Bodipy^+^ uEV concentration and multiple patient clinical parameters was assessed (**Supplemental Figure 12**). In addition, there was no significant correlation between patient age or creatinine concentration and Bodipy^+^ uEV concentration (**Supplemental Figure 13**). An average of 69.5% of the Bodipy^+^ uEVs were also positive for the exosome marker, tetraspanin CD63 (**Figure 5E**). The fluorescently tagged uEVs could be visualized using the ImageStream^®^ inbuilt microscope to ensure that aggregates, doublets or background/aberrant fluorescence were not being analyzed (**Figure 5F**).

### uEV-Derived miR-155 and Surface Markers Are Altered During Progression of CAD

**Figure 6:**
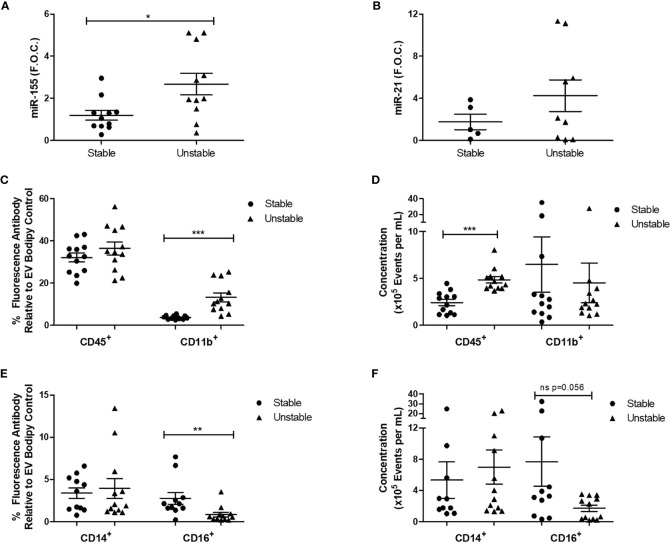
Patients with unstable CAD have increased uEV miR-155 expression and alterations in CD45^+^, CD11b^+^, and CD16^+^ uEVs. For qRT-PCR analysis, four EV pellets (derived from 6-ml urine) were re-suspended in TRIzol™ for RNA extraction. 100ng of RNA was reverse transcribed to cDNA and qRT-PCR analysis was performed for **(A)** miR-155 (n = 11 per group) and **(B)** miR-21 (5 stable and 9 unstable CAD patients) expression using the reference genes, RNU6B and RNU48. For flow cytometry analysis, four pellets were re-suspended in 300 μl of sterile-filtered PBS, 50 µl of each sample was stained with the membrane stain, Bodipy and the antibodies CD45, CD11b, CD14, and CD16 (n = 1 per group). Ten thousand EVs positive for Bodipy were analyzed. **(C)** The percentage of CD45 and CD11b fluorescence antibody relative to Bodipy stain was analyzed. **(D)** The concentration of CD45 and CD11b fluorescent EVs per ml was analyzed. **(E)** The percentage of CD14 and CD16 fluorescence antibody relative to Bodipy stain was analyzed. **(F)** The concentration of CD14 and CD16 fluorescent EVs per ml was analyzed. Statistical analysis was performed using non-parametric Mann-Whitney U tests where *p < 0.05, **p < 0.01, and ***p < 0.001 represent statistical significance. Significance between groups is indicated by capped lines.

uEV-derived miR-155 and another microRNA associated with inflammation, miR-21, were analyzed to investigate their correlation with disease progression. qRT-PCR analysis showed that there was a significant increase in miR-155 expression in uEVs isolated from unstable CAD patients compared to those from stable CAD patients (stable 1.19 ± 0.2 vs. unstable 2.67 ± 0.5 fold change, *p < 0.05) (**Figure 6A**) while miR-21 expression was unchanged (**Figure 6B**).

Given that the concentration of uEVs and uEV miR-155 expression were altered in unstable CAD patients in comparison to stable CAD patients, uEVs were further analyzed by flow cytometry to examine specific surface markers related to immune cells (CD45, CD14, CD16) and adhesion (CD11b). CD11b is an integrin that facilitates cell adhesion and has previously been used in combination with other markers to characterize leukocyte derived EVs isolated from plasma (36). The percentage of CD11b^+^ EVs was increased in unstable CAD patients compared to the stable population (stable 3.71 ± 0.24% vs. unstable 13.20 ± 2.12% fluorescence, ***p < 0.001) (**Figure 6C**). CD45, a leukocyte marker previously shown to be detectable on EVs derived from urine (49) and plasma (50) was also analyzed. Although the percentage of fluorescent CD45^+^ uEVs was unchanged (**Figure 6C**), the concentration of CD45^+^ uEVs was significantly increased in the unstable CAD patients (stable 2.4 × 10^5^ ± 3.3 × 10^4^ events/ml vs. unstable 4.8 × 10^5^ ± 3.5 × 10^4^ events/ml, ***p < 0.001) (**Figure 6D**). CD16 and CD14 can be used to detect plasma EVs (51). CD16 is a type 1 transmembrane receptor that is expressed on multiple blood cells including tissue macrophages (52) and subsets of monocytes (53). Unstable CAD patients had a significantly reduced percentage of CD16^+^ uEVs when compared to stable patients (stable 2.76 ± 0.7% vs. unstable 0.84 ± 0.27% fluorescence, **p < 0.01) (**Figure 6E**). Although there was also a trend toward decreased CD16^+^ uEV concentration, this was not statistically significant (**Figure 6E**). CD14 was unchanged between the two groups (**Figures 6E, F**).

## Discussion

This study presents novel evidence linking miR-155 biology with the progression and regression of atherosclerosis. Herein, we have shown an inverse association between miR-155 and plaque regression in a murine model of atherosclerosis. Furthermore, this microRNA has downstream cellular effects that suppress anti-inflammatory signaling proteins. Moreover, in this human clinical study, uEV-derived miR-155 was associated with plaque instability (manifested as patients presenting with unstable CAD), suggesting a role for miR-155 as a biomarker and/or therapeutic target.

CLA, a pro-resolving lipid mediator, has previously been shown to attenuate progression and induce regression of atherosclerosis *via* regulation of monocyte function and phenotype (3–5). However, the molecular mechanisms underlying the atheroprotective effect of CLA have not been fully elucidated. *In silico* analysis of transcriptomic data from the aorta of ApoE^−/−^ mice supplemented with CLA identified miR-155 to be among the 12 upstream microRNA networks predicted to be inhibited by CLA. Previously, it has been shown that miR-155 promotes macrophage inflammation (19, 24), is increased in atherosclerotic plaques (46) and in pro-inflammatory macrophages involved in the pathogenesis of the disease (20). Thus, using our established murine model of atherosclerosis regression we investigated the effects of CLA on miR-155 expression. The aortic specimens retained from a previously published CLA-regression study were analyzed (5). Similarly to previously published studies (22, 26, 54) we showed that miR-155 expression is increased in aorta from ApoE^−/−^ mice on a 1% CD, shown to induce atherosclerotic lesion development which was coincident with an increase in the pan-macrophage marker, CD68. We found that aortic miR-155 expression was downregulated following 4 weeks of CLA supplementation. We have previously shown that there was increased IL-10 signaling, decreased TNF-α and increased numbers of anti-inflammatory monocytes in these aortae from CLA-supplemented mice (5). Further analysis of aortae from CLA-supplemented mice showed a trend toward increased BCL-6 expression coincident with decreased miR-155. BCL-6 has been previously shown to be upregulated in miR-155^−/−^/ApoE^−/−^ mice following 24 weeks of high CD and miR-155 was shown to regulate efferocytosis *via* regulation of BCL-6 (22). Here we show that CLA, which induced regression of pre-established atherosclerosis, inhibits miR-155 expression *in vivo*.

Given that this reduction in aortic miR-155 expression following CLA supplementation coincided with a reduction in TNF-α expression and CD68^+^ macrophages (3, 5), which express miR-155, further experiments were designed to investigate the direct regulatory effects of CLA on miR-155. CLA treatment decreased miR-155 expression in both M1-like and M2-like polarized THP-1 macrophages. Indeed the CLA mediated downregulation of miR-155 in M2 macrophages suggests that these may have reduced capacity to adopt an M1-like phenotype as previously it has been shown that overexpression of miR-155 in M2 BMDMs polarized macrophages toward an M1-like phenotype (55). Coincident with the CLA-mediated decrease in miR-155 expression, there was an increase in protein expression of miR-155 targets, BCL-6 and SHIP-1, in M2 macrophages. In addition, there was a significant increase in the anti-inflammatory marker, MR, in M2 macrophages. Previously, we demonstrated that CLA induced MR and CD163 expression in human peripheral blood monocytes and macrophages (Gaetano et al., 2015). Furthermore, BMDMs from CLA-fed mice stimulated with LPS had increased M2 marker expression including Arginase-1 and MR (5). Collectively these findings show that CLA shifts the macrophage toward an anti-inflammatory phenotype and this is coincident with suppression of miR-155.

The exact mechanisms by which CLA blend suppresses miR-155 remain to be elucidated. McCarthy et al. demonstrated that CLA supplementation, increased expression of IL-10, IL-10 Receptor 1, IL-10 Receptor 2, IL-1 Receptor Antagonist, and p-STAT-3 signaling. The IL-10 signaling pathway has been shown to inhibit miR-155 in the presence of LPS *via* a STAT-3 and ETS1 dependent mechanism resulting in an increase in SHIP-1 expression (56). Therefore, we postulated that CLA supresses miR-155 *via* upregulation of the STAT-3/IL-10 signaling pathway, however, IL-10 and p-STAT-3 were unchanged by CLA in polarized macrophages. However, we also showed that CLA upregulates p-Akt expression in M1-like macrophages which may also impact on the suppression of miR-155. Previous work has shown that Akt1^−/−^ macrophages have increased levels of miR-155 while viral transfection of BMDMs with Akt reduced miR-155 in the presence of LPS (57). In conclusion, it remains to be elucidated whether the effects of CLA on miR-155 are essential for induction of an M2-like macrophage and regression of atherosclerosis.

To understand the inflammatory effects of miR-155 upregulation we transfected THP-1 M0 macrophages with miR-155 mimic. Although the miR-155 mimic did not increase p-STAT-1 pro-inflammatory signaling, it did decrease the anti-inflammatory proteins, BCL-6 and p-STAT-3, in macrophages. This is in keeping with the previous studies which showed that miR-155 mediated its pro-atherogenic and pro-inflammatory effects in part *via* suppression of BCL-6. Unstimulated miR-155*^−/−^* BMDMs had increased levels of BCL-6 and silencing of BCL-6 in these miR-155^−/−^ BMDMs increased C-C Motif Chemokine Ligand 2 (CCL2) and TNF-α secretion. Furthermore, silencing of BCL-6 *in vivo* resulted in increased lesion area, increased CCL2 positive macrophages and increased number of lesional macrophages (19). While this previous work was in murine models, here we provide translational relevance to this pathway by using human THP-1 macrophages.

Macrophage deletion of STAT-3 followed by LPS stimulation has been shown to result in production of pro-inflammatory cytokines (58). We have shown here that miR-155 mimic inhibits STAT-3 phosphorylation in LPS-stimulated macrophages which may allow for increased pro-inflammatory cytokine production. This suppression of p-STAT-3 is likely mediated *via* an indirect mechanism, as to date STAT-3 has not been defined as a miR-155 target. Interestingly, STAT-3 expression and activation mediates the effects of IL-10 signaling, shown to reduce atherosclerotic lesion size (59) and promote the induction of an M2 anti-inflammatory phenotype (58). Therefore, given that miR-155 is a negative regulator of anti-inflammatory signaling, we hypothesize that CLA mediates its atheroprotective effect in part *via* suppression of miR-155 expression. In addition, BCL-2, an anti-apoptotic protein and validated miR-155 target, was suppressed by the miR-155 mimic suggesting that miR-155 may increase the apoptotic capacity of the macrophage. This is in keeping with other studies which showed the regulatory effects of miR-155 on BCL-2 and cardiac apoptosis in a murine model of LPS-induced cardiac dysfunction (60). miR-155 inhibition prevented the downregulation of H_2_O_2_ induced BCL-2 in cardiomyocytes and was protective against ischemia-induced apoptosis *in vivo* (61). In contrast, BMDMs lacking miR-155 were more susceptible to apoptosis during infection, suggesting that miR-155 has an anti-apoptotic function (62). Notably, miR-155 regulates multiple cell death pathways, as macrophage derived miR-155 containing exosomes enhanced pyroptosis in cardiomyocytes through the direct targeting of FoxO3a (63). Human monocyte derived foam cells, obtained after prolonged exposure to oxidized LDL [a known inducer of miR-155 expression (46, 64)], respond to inflammasome activation preferentially with pyroptosis combined with other types of necrotic death (65). Thus, it could be speculated that switching between cell death pathways is an effector mechanism of miR-155 as these pathways have the capacity to regulate the immunogenic cell death burden which can contribute to atherosclerosis development.

Given that miR-155 was altered during murine regression of atherosclerosis we sought to further investigate alterations in miR-155 in atherosclerotic disease progression and to characterize the uEV signature; from a stable CAD diagnosis to the occurrence of a myocardial infarction or unstable angina (unstable CAD). Previously we have shown that there is increased expression of pro-inflammatory M1 markers in CEA samples from patients who had experienced ischemic events. Furthermore, M2 markers were localized to stable plaque regions and were inversely associated with disease progression (66). Further understanding of the changes in biofluid-derived EVs during disease progression could be useful in the future to stratify patient risk and select the most appropriate therapeutic intervention.

Isolation of uEVs was performed using medium speed centrifugation which has been used in other studies (49, 67). In accordance with the 2018 MISEV guidelines (68) EVs were extensively characterized by TEM, NTA, and flow cytometry. This centrifugation method isolates a heterogenous EV population and TEM showed vesicles in the 100-nm size range that had the characteristic round cup shape induced by the fixation process and were similar to EVs from mouse dendritic cells observed by Théry et al. (69). NTA further demonstrated that the highest concentrations of particles detected were within the 100–150 nm size range; these events corresponded to intact uEVs, as shown by flow cytometry analyses. In addition, these uEVs expressed the exosome marker, tetraspanin CD63.

miR-155 expression was increased in uEVs from the unstable CAD patients when compared to stable CAD patients. This corresponds to previous studies analyzing microRNAs in circulation where miR-155 was shown to be increased in plasma from patients with atherosclerosis when compared to normal controls (46). Plasma EVs from acute CAD patients had increased levels of several inflammatory microRNAs including miR-155, miR-21 and miR-146 when compared to plasma EVs from stable CAD patients (70). In addition, blood-derived miR-155 has been shown to demonstrate a significant discriminatory power to predict the presence of thin-cap fibroatheromas (71).

In this study we noted a decrease in uEV concentration from patients with unstable CAD which could be a consequence of multiple factors which may be affected by CAD progression such as EV biogenesis, secretion and uptake. Patients with diabetes and hypertension, two common comorbidities observed in a CAD population, have previously been shown to have an altered uEV profile (72–75). Notably, incipient diabetic nephropathy was associated with alterations in four microRNAs derived from uEVs (76). In addition, anti-hypertensives alone and in combination with statin therapy can also affect the circulating EV population (77, 78). In this study, although patients with hypertension and type 2 diabetes had a decreased uEV concentration, this was not significant. The diagnosis of unstable CAD was the primary contributing factor associated with a significant decrease in uEV concentration.

The surface of these uEVs was positive for several markers of cells of the innate immune system including CD14, CD16, and CD45. CD45 is highly expressed on mature leukocytes and is frequently used as a leukocyte marker (79). Given the mechanisms of EV biogenesis, these CD45^+^ EVs are likely to be EV-derivates from their parent leukocyte cells. The concentration of CD45^+^ EVs was increased in the unstable cohort and we postulate that this is due to the reactivity of the unstable plaque and/or the occurrence of a previous acute coronary event. Indeed it has previously been demonstrated that CD3^+^/CD45^+^/Annexin V^+^ (lymphocyte-derived) plasma EVs were increased in individuals who had suffered a cardiovascular event (50).

In contrast to what was observed with CD45, CD16 was significantly decreased in unstable CAD patient uEVs. CD14^++^CD16^−^ classical monocytes, have increased CCR2 and IL-10 expression (80) while non-classical CD14^+^CD16^++^ monocytes express CX_3_CR1 which facilitates migration and produce high levels of pro-inflammatory cytokines TNF-α, IL-1β, and IL-6 (81). A prospective study by Berg et al. showed that CD14^++^CD16^−^ monocytes were increased at baseline in individuals that later went on to suffer an ischemic event in comparison to the control stable CAD group. Furthermore, CD16^+^ monocytes were negatively correlated to the extent of carotid atherosclerosis and were demonstrated to predict future cardiovascular risk (82). Thus given the predictive power of CD16 on monocytes and the decreased expression of CD16^+^ uEVs in the unstable CAD population observed here, we postulate that despite already having experienced a cardiac event unstable CAD patients are still at greater risk of an event in comparison to the stable cohort. However, the origin and prognostic potential of these uEVs needs to be definitively shown.

CD11b was significantly increased in the uEVs derived from unstable CAD patients. CD11b, the alpha chain (Mac-1 alpha), combines with CD18 to form the integrin macrophage receptor 1 (Mac-1), a leukocyte-specific integrin. CD11b has been previously identified on exosomes derived from murine dendritic cells by proteomic analysis and flow cytometry (83). Mac-1 facilitates adherence of neutrophils and monocytes to the endothelium. The function of CD11b on EVs is unknown although previously EV adhesion was blocked using an anti-CD18 antibody (84). Further investigation is required to determine if the decrease in uEV concentration in the unstable CAD patients is due to the increased CD11b on EVs thus facilitating binding to ligands on endothelium and subsequent EV uptake and removal from circulation.

A recent proteomics study has shown that uEV proteins are derived from a broad range of organs and not enriched in renal proteins. Furthermore, heterogenous uEVs that sample from across the body are stable within individuals over time. Interestingly, this study also showed that uEVs have potential as biomarkers for neurological diseases such as Parkinson’s disease (40). Therefore, given that urine collection is a non-invasive procedure and the unrestricted free access of EVs in circulation, uEVs may have potential as biomarkers for a broad range of diseases.

In summary, we have shown that CLA decreases miR-155 in human THP-1 polarized macrophages and in murine aortae in an *in vivo* model of atherosclerosis regression. In addition, miR-155 was increased in uEVs from unstable CAD patients. Notably, as CAD progressed there was a decreased uEV concentration, increased CD45^+^ and CD11b^+^ uEVs, and decreased CD16^+^ uEVs. These findings indicate that miR-155 is associated with disease progression and regression and that uEVs and miR-155 expression could be further exploited as potential prognostic indices of disease progression and severity.

## Author’s Note

This work has been submitted in part in a PhD thesis, to University College Dublin by SF (January 2020).

## Data Availability Statement

The raw data supporting the conclusions of this article will be made available by the authors, without undue reservation.

## Ethics Statement

The studies involving human participants were reviewed and approved by the Ethics Committee of The Mater Misericordiae University Hospital, Dublin, Ireland and the Ethics Committee of St. Vincent’s University Hospital, Dublin, Ireland. The patients/participants provided their written informed consent to participate in this study. The animal study was reviewed and approved by the Animal Research Ethics Committee (AREC) at University College Dublin (Study approval code AREC P-08-62).

## Author Contributions

SF, RB, CM, MS, EB, and OB designed the experimental work performed. CM carried out the *in vivo* experiments. SF carried out the *in vitro* and *ex vivo* experiments. NM, NR, and MB were responsible for participant recruitment and sample collection of urine and atherosclerotic plaque samples. SF carried out all analysis on clinical samples. MP, SO and MS provided expertise and protocols for flow cytometry experiments and analysis. All figures were prepared by SF. SF and OB wrote and revised the manuscript. All authors contributed to the article and approved the submitted version.

## Funding

The work outlined in this paper was funded by the Irish Research Council to SF and OB. Project Title: Investigation of the microRNA signature associated with regression of atherosclerosis, Project ID: GOIPG/2015/3435. This work was also funded by UCD Wellcome Institutional Strategic Support Fund, jointly supported by UCD and the SFI-HRB Wellcome Biomedical Partnership to OB. Extracellular vesicle flow cytometry work was funded by the EVOluTION programme which received funding from the European Union’s Horizon 2020 research and innovation programme under the Marie Sklodowska-Curie grant agreement No. 675111 (SO and MP). The ImageStream™X used was funded by the Wellcome Trust (infrastructure grant 101604/Z/13/Z). Flow cytometry work was funded by grants awarded to MS from the German Research Foundation (DFG; STR 1570/1-1) and the Braun Foundation (Braun; BBST-D-18-00018).

## Conflict of Interest

The authors declare that the research was conducted in the absence of any commercial or financial relationships that could be construed as a potential conflict of interest.

## Abbreviations

ApoE, apolipoprotein E; BCL, B-cell lymphoma; *BIC*, B-cell integration cluster; BMDM, bone marrow-derived macrophage; CAD, Coronary Artery Disease; CLA, Conjugated linoleic acid; CVD, Cardiovascular disease; dH_2_O, Deionized water DMSO, Dimethyl sulfoxide; ELISA, Enzyme-linked immunosorbent assay; EV, Extracellular vesicle; GAPDH, glyceraldehyde 3-phosphate dehydrogenase; HLA-DR, human leucocyte antigen DR; IL, Interleukin; IFN-γ, Interferon gamma; LDL, Low density lipoprotein; LPS, Lipopolysaccharide; miR, microRNA; miR-155, microRNA-155-5p (Guide Strand); *MIR155HG*, MIR155 host gene; ND, Normal chow diet; ns, not significant; NTA, Nanoparticle tracking analysis; PBS, phosphate buffered saline; p, phosphorylated; PMA, phorbol 12-myristate 13-acetate; qRT-PCR, quantitative real-time PCR; RIPA, Radioimmunoprecipitation assay; ROS, Reactive Oxygen Species; RPMI, Roswell Park Memorial Institute; SDS-PAGE, sodium dodecyl sulphate-polyacrylamide gel electrophoresis; SEM, standard error of the mean; SHIP-1, Src homology 2 domain-containing inositol 5’-phosphatase 1; SOCS1, Suppressor Of Cytokine Signaling 1; STAT; Signal transducer and activator of transcription; STEMI, ST-elevated myocardial infarction; TEM, Transmission electron microscopy; TNF-α, tumor necrosis factor-alpha; uEV, Urinary extracellular vesicle; 1%CD, 1% Cholesterol diet; 1% CD+ CLA, 1% Cholesterol diet + Conjugated linoleic acid; ^−/−^, Knockout.
