# Supplementary material for: microRNA-155 Is Decreased During Atherosclerosis Regression and Is Increased in Urinary Extracellular Vesicles During Atherosclerosis Progression
Source: Front Immunol. 2020 Dec 17;11:576516. doi: 10.3389/fimmu.2020.576516 (PMC7773661; doi:10.3389/fimmu.2020.576516)
Supplement: Supplementary file 1 [file DataSheet_1.docx]

**Supplemental Methods**

**Table 1: Flow cytometry antibodies and EV stains used.** Conjugates used include, Allophycocyanin (APC), Phycoerythrin (PE), Fluorescein Isothiocyanate (FITC), Texas Red.

| **Antigen** | **Conjugate** | **Conc.** | **Clone** | **Company** | **Catalogue** |
| --- | --- | --- | --- | --- | --- |
| **HLA-DR** | FITC | 1μg/mL | TU39 (RUO) | BD Bioscience | 555558 |
| **MR** | APC | 2μg/mL | 15.2 | BD Bioscience | 321110 |
| **CD11b** | APC | 10 μg/mL | ICRF44 | Biolegend | 301316 |
| **CD14** | APC | 2 μg/mL | 61D3 | Biolegend | E120750149120 |
| **CD16** | PE | 0.6μg/mL | CBC16 | Biolegend | 12016842 |
| **CD45** | PE | 0.12 μg/mL | 2D1 | Biolegend | 12945942 |
| **CD63** | FITC | 1mg | 805 | BioRad | MCA2142F |
| **Bodipy** | FITC | N/A | N/A | ThermoFisher | B10250 |
| **Bodipy** | Texas Red | N/A | N/A | ThermoFisher | D6116 |

**Table 2: Primary antibodies and dilutions used for Western blotting.**

| **Antibody** | **Company** | **Catalogue** | **Dilution** |
| --- | --- | --- | --- |
| **β-Actin (C4)** | Santa Cruz | SC-47778 | 1:2,000 |
| **BCL-2** | Santa Cruz | SC-7382 | 1:1,000 |
| **BCL-6** | Santa Cruz | SC-7388 | 1:500 |
| **CD9** | System Biosciences | EXOAB-CD9A-1 | 1:500 |
| **CD63** | System Biosciences | EXOAB-CD36A-1 | 1:1,000 |
| **CD68** | Santa Cruz | SC-20060 | 1:500 |
| **CD81** | System Biosciences | EXOAB-CD81A-1 | 1:500 |
| **GAPDH (D16H11)** | Cell Signalling | 5174 | 1:5,000 |
| **HSP70** | System Biosciences | EXOAB-Hsp70A-1 | 1:500 |
| **IL-1RA (20D8)** | Cell Signalling | 3865 | 1:1,000 |
| **MR** | Cell Signalling | 12981 | 1:500 |
| **Phosphorylated Akt (Ser473)** | Cell Signalling | 9271 | 1:2,000 |
| **Phosphorylated STAT-1**  **(S727)** | Cell Signalling | 9177 | 1:1,000 |
| **Phosphorylated STAT-3 (Y705)** | Cell Signalling | 9145 | 1:1,000 |
| **SHIP-1 (D1163)** | Cell Signalling | 2728 | 1:500 |
| **STAT-1 (D1K9Y)** | Cell Signalling | 14994 | 1:2,000 |
| **STAT-3 (79D7)** | Cell Signalling | 4904P | 1:2,000 |
| **VCAM-1** | Santa Cruz | SC-13160 | 1:500 |

**Table 3: Taqman probes used for RT-PCR.** The table includes Taqman Probe name, Full gene name, Aliases of the gene, the HGNC ID of the gene and the Assay ID.

| Taqman Probe | Full Gene Name | Alias | HGNC ID | Assay ID |
| --- | --- | --- | --- | --- |
| hsa-miR-21-5p | microRNA 21 | miR-21 | 31586 | 000397 |
| hsa-miR-155-5p | microRNA 155 | miR-155 | 31542 | 002623 |
| mmu-miR-155-5p | microRNA 155 | miR-155 | 31542 | 002571 |
| RNU6B | RNA, U6 small nuclear 6, pseudogene | RNU6-6P | 10228 | [001093](https://www.thermofisher.com/order/genome-database/details/microrna/001093?CID=&ICID=&subtype=) |
| U6 snRNA | U6 snRNA biogenesis phosphodiesterase 1 | USB1 | 25792 | 001973 |
| 18S rRNA | 18S ribosomal RNA | 18S rRNA | 1383 | Hs99999901_s1 |
| BCL-6 | BCL6 transcription repressor |  | 1001 | Hs00153368_m1  Mm00477633_m1 |
| INPP5D | Inositol Polyphosphate-5-Phosphatase D | SHIP-1 | 6079 | Hs00183290_m1  Mm00494987_m1 |
| GAPDH | Glyceraldehyde-3-phosphate dehydrogenase | GAPD | 4141 | Hs99999905_m1  Mm99999915_g1 |
| MRC1 | Mannose receptor C-type 1 | CD206,  MR | 7228 | Hs00267207_m1 |
| CCL2 | C-C motif chemokine ligand 2 | MCP-1 | 10618 | Hs00234140_m1 |

**Supplemental Method 1: Transfection of miR-155 into human plaque *ex vivo***

The Ethics Committee of St Vincent’s University Hospital, Dublin, Ireland approved the collection of patient atherosclerotic plaque tissue for research purposes. The study was performed in accordance with institutional guidelines and in compliance with international laws. All patients undergoing CEA gave written informed consent and the study was performed in accordance with the World Medical Association’s Declaration of Helsinki. Following surgical removal from patients (n=5, age: 67.5±4.3), plaques were placed in phosphate buffered saline (PBS) (Gibco, Thermo Fisher Scientific, UK). Plaques were further sectioned into cubes (approximately 0.3cm^3^ in size) and 2 cubes per well were added to a 24 well plate. Plaque sections were treated with hsa-miR-155-5p miRVana® miRNA mimic (20nmol/L) (Ambion, Thermo Fisher Scientific, UK) using Lipofectamine^TM^ 2000 (0.1%) (Invitrogen, Thermo Fisher Scientific) and Opti-MEM I media (10%) (Gibco, Thermo Fisher Scientific, UK) diluted in 450μL serum-free RPMI per well in a 24-well plate. Plaques were transfected for 18hr and incubated at 37 °C in a humidified atmosphere of 95% air and 5% CO_2_, the plaque pieces were then treated with LPS (1μg/mL) for a further 6hr using deionised water (dH_2_O) as the control as previously described ([Brennan, 2017](#_ENREF_5)). Plaque tissue underwent a series of homogenization steps (Supplemental Methods Table 4) using a TissueLyser II (Qiagen Ltd, Manchester, UK) and RNA was extracted using TRIzol^TM^ Reagent (Ambion, Thermo Fisher Scientific, UK) as per manufacturer’s instructions.

**Table 4:** **Plaque homogenization process using TissueLyser II and TRIzol^TM^.**

| **Steps** | **Method** | | **Speed/Frequency** | **Time (min)** |
| --- | --- | --- | --- | --- |
| 1 | | Add 100uL TRIzol + 1 steel bead +Plaque Tissue | 30 | 10 |
| 2 | | Add 400uL TRIzol | 30 | 10 |
| 3 | | Repeat Step 2 if not homogenized | 30 | 10 |
| 4 | | Centrifuge TRIzol Supernantant | 12,000 rpm | 10 |
| 5 | | Discard pellet + perform TRIzol Extraction as per manufacturers’ instructions | | |
