# Supplementary material for: microRNA-155 Is Decreased During Atherosclerosis Regression and Is Increased in Urinary Extracellular Vesicles During Atherosclerosis Progression
Source: Front Immunol. 2020 Dec 17;11:576516. doi: 10.3389/fimmu.2020.576516 (PMC7773661; doi:10.3389/fimmu.2020.576516)
Supplement: Supplementary file 2 [file DataSheet_2.docx]

**Supplemental Figures**


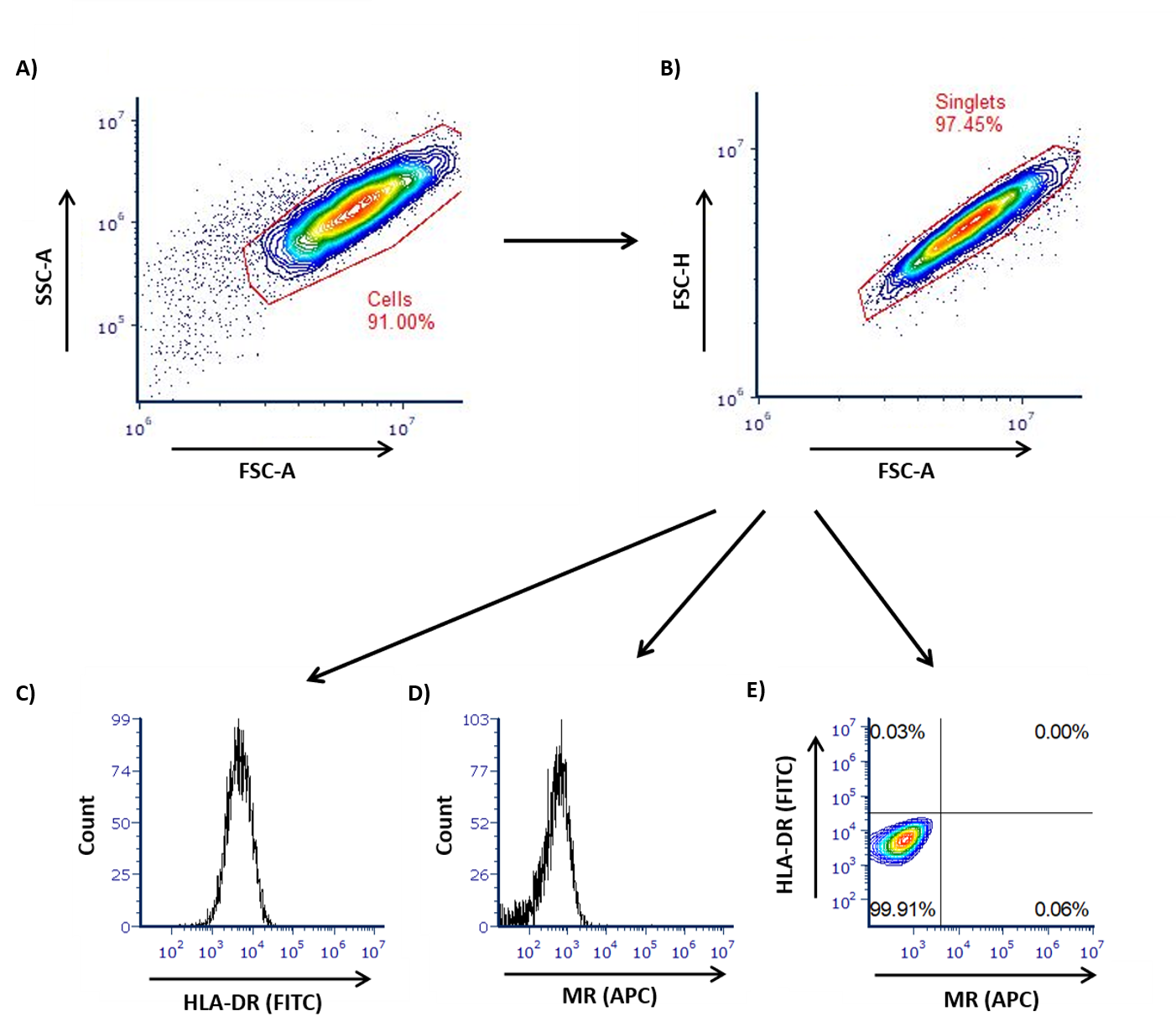


**Supplemental Figure 1: Gating strategy used in flow cytometry analysis to detect baseline fluorescence of HLA-DR (FITC) and MR (APC) on unlabelled untreated macrophages. Using the Accuri C6 Flow Cytometer** viable cells were gated on a forward scatter (FSC) and side scatter (SSC) plot to exclude cell debris **(A)**. Viable cells were then further gated on a forward scatter area (FSC-A) and forward scatter height (FSC-H) plot to determine the singe cell population (singlets) **(B)**. Fluorescence signal was analyzed using the 3 blue, 1 red laser configuration and the FL-1 (filter wavelength: 533/30; FITC signal) and FL-4 (filter wavelength: 675/25; APC signal) channel of the Accuri C6. To define the negative cell populations, the unlabelled single cell population was then analyzed to detect the baseline fluorescence intensity for HLA-DR(FITC) **(C)** and MR-(APC) **(D)**. Quadrant regions were established with the unlabelled, untreated macrophages assigned as the cell population negative for HLA-DR (FITC) and MR (APC) **(E)**. Conjugates used were allophycocyanin (APC) and fluorescein isothiocyanate (FITC). Graphs were generated using FCS Express 6 Software.


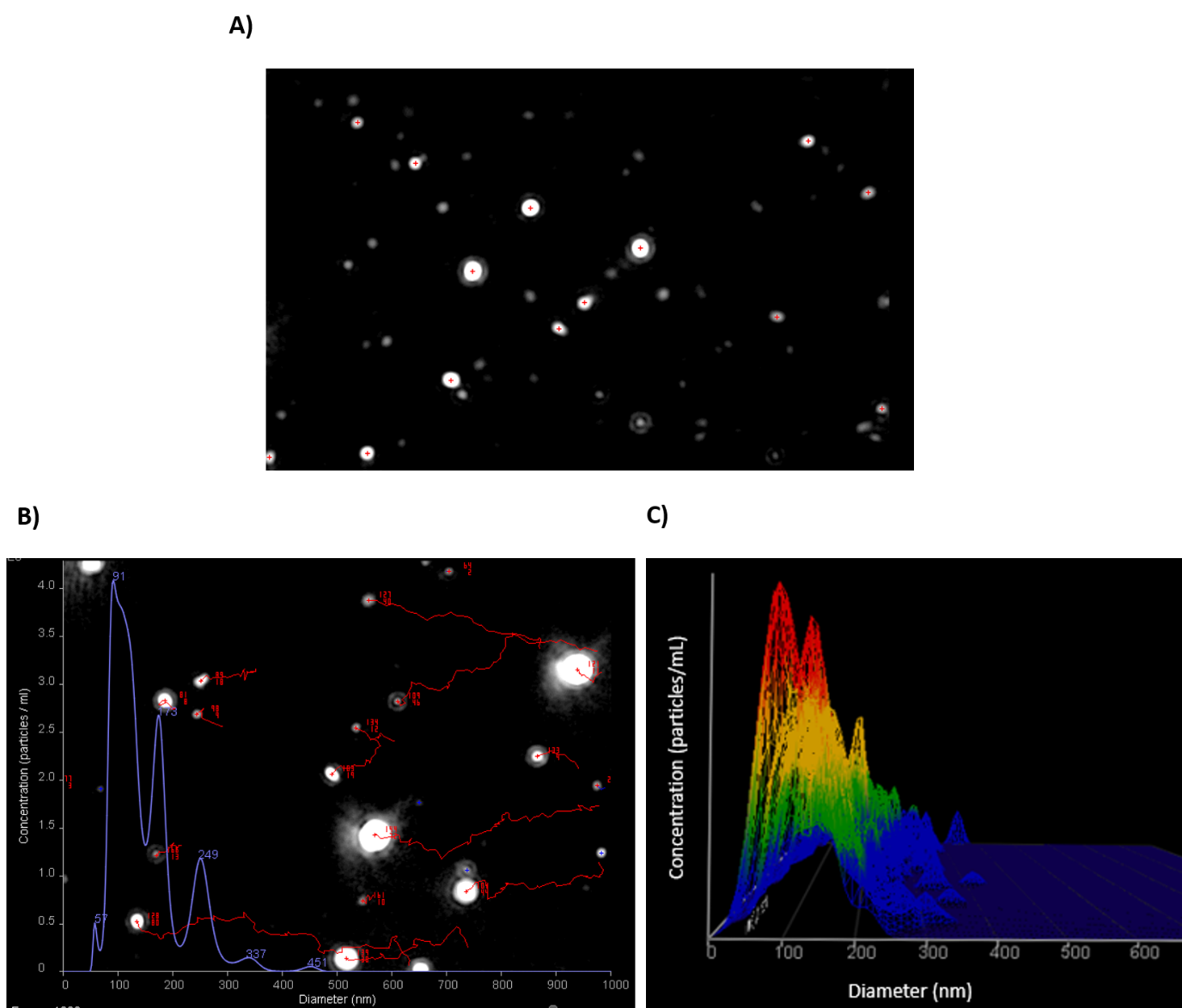


**Supplemental Figure 2: NanoSight NS300 representative images of nanoparticle tracking analysis on urinary EVs.** A laser beam is used to analyze the particles as they flow through the NanoSight flow chamber and the microscope captures and magnifies the scattered light. **(A)** A still image from a video file taken from a camera attached to the microscope which captures the particles moving under Brownian motion. In the representative image shown, the white dots are particles in suspension and the detection threshold has been set to only analyze the dots depicted with a red cross. **(B)** All videos are analyzed and each particle is tracked and analyzed by software using the Stokes Einstein equation to determine the size distribution. The concentration of the particles in each sample is also determined. **(C)** A representative 3-dimensional plot showing the diameter, concentration and intensity (not shown) that is generated during analysis of video files. Images were obtained from the Nanosight NS300.

**
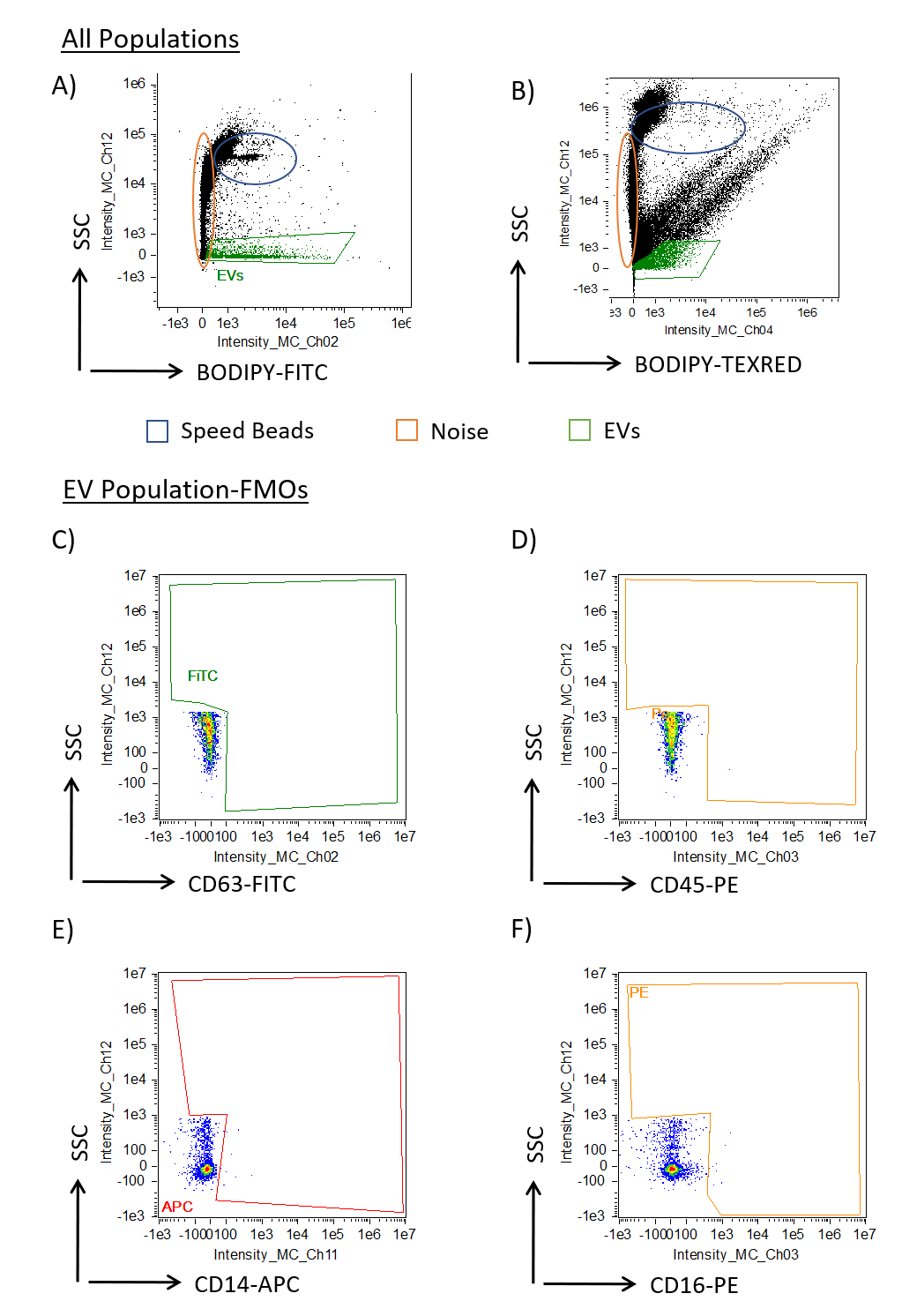
**

**Supplemental Figure 3: Flow cytometry gating strategy and FMO controls on uEVs from patients with CAD.** Following centrifugation, four pellets were re-suspended in 300μL of sterile filtered PBS for flow cytometry using an ImageStream^TM^X Mark II Flow Cytometer. 50µL of each sample was stained with Bodipy, and the EVs were analyzed using an ImageStream^TM^X Mark II Flow Cytometer. All graphs show the side scatter channel (SSC) on the y-axis and fluorescent intensity on the x-axis. Flow cytometry gates show the detection of fluorescent EVs tagged with **(A)** Bodipy tagged FITC and **(B)** Texas Red. Gates were established to selectively gate on the uEV population which is characterized as having low side scatter and low to medium fluorescent intensity. **(C-F)** FMO controls were established to ensure accurate gating on samples. Conjugates used include, Allophycocyanin (APC), Phycoerythrin (PE), Fluorescein Isothiocyanate (FITC) and Texas Red.


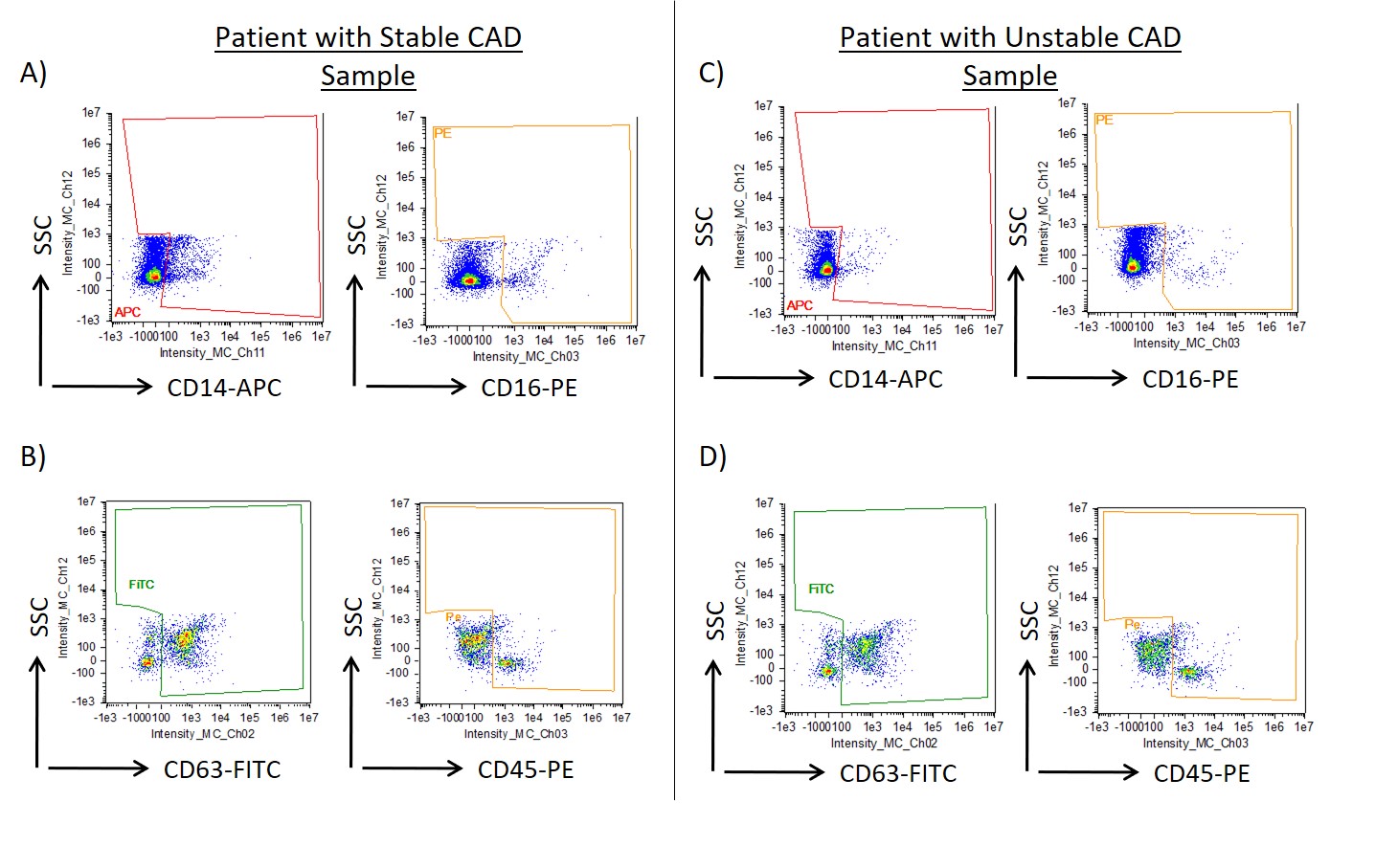


**Supplemental Figure 4: Flow cytometry gating strategy sample graphs from analysis of uEVs from a patient with stable CAD and a patient with unstable CAD.** 50µL of each uEV sample was stained with Bodipy, and the antibody combination of CD14 and CD16 or CD63 and CD45, the EVs were then analyzed using an ImageStream^TM^X Mark II Flow Cytometer. Flow cytometry graphs show the gating strategy for the following antibodies and their fluorescent conjugates CD14-APC, CD16-PE, CD63-FITC and CD45-PE. **(A-B)** Graphs are representative of uEVs from a patient with stable CAD. **(C-D)** Graphs are representative of uEVs from a symptomatic CAD patient. Flow cytometry gates are based on FMO controls and visual assessment of fluorescent uEVs using the inbuilt microscope. Increased antibody detection events, visualized here as coloured dots occurring inside the drawn gate, are indicative of presence of that antibody marker on a Bodipy^+^ uEV.


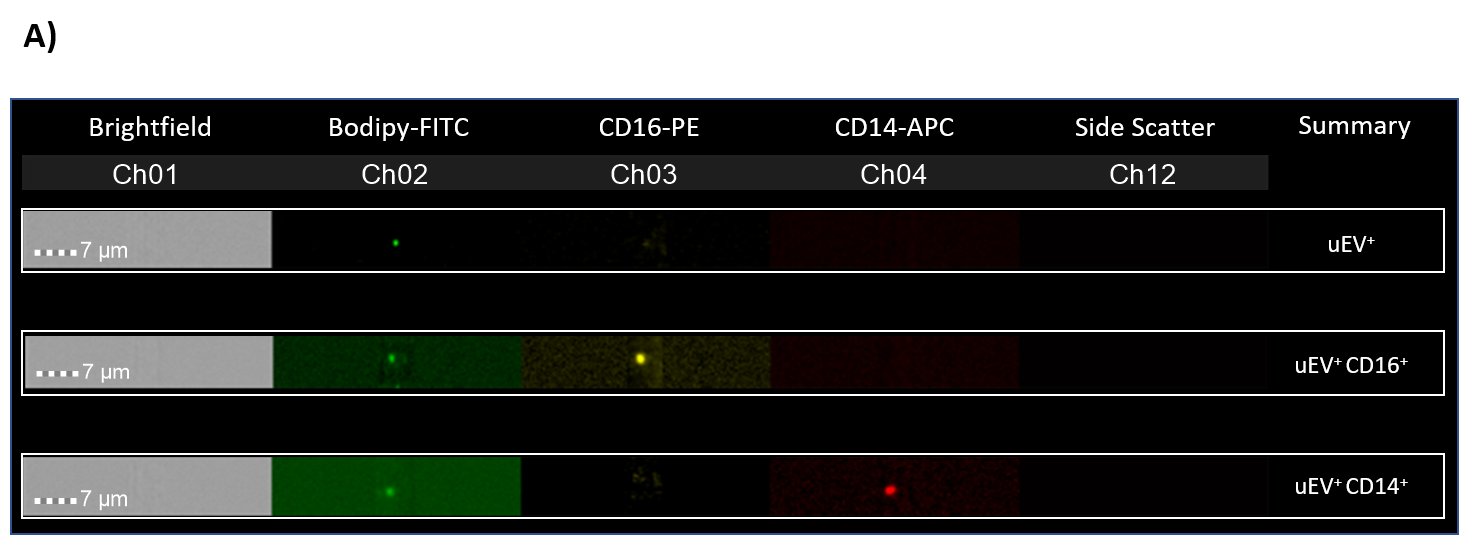


**Supplemental Figure 5: Images of Bodipy^+^, CD14^+^ and CD16^+^ uEVs.** EVs were isolated from 6mL of urine using a benchtop centrifugation protocol at 4 °C. Four EV pellets were re-suspended in 300µL PBS. 50µL was then stained with Bodipy and the respective antibodies and then analyzed by flow cytometry using the ImageStream^TM^X Mark II. Images of single stained uEVs were obtained using the inbuilt microscope which allowed assessment and exclusion of doublets, or any aberrant fluorescent background. The Bodipy membrane stain was used to identify uEVs. Images of uEVs stained with Bodipy and positive for specific fluorescent antibodies CD14 and CD16. Conjugates used include, allophycocyanin (APC), phycoerythrin (PE) and fluorescein isothiocyanate (FITC).

| **Upstream Regulator** | **Predicted Activation State** | **Activation z-score** | **p-value of overlap** | **Target molecules in dataset** |
| --- | --- | --- | --- | --- |
| miR-30c-5p | Inhibited | -3.073 | 0.00142 | ADAMTS15, ATRX, Dst, GALNT1,  GPD2, IL6, NCEH1, NID1, NPR3,  PLEC, PTPRK, RAD23B, TNRC6A |
| let-7 | Inhibited | -2.792 | 0.00000522 | ACVR1C, ADRB1, BCAT1, CCNA2,  CD44, CDC20, CDCA5, CDCA8,  CDK1, CDT1, DBF4, E2F5, EIF4A1,  ERO1A, IL6, LRIG1, MCM3, MCM5,  PA2G4, PNKD, RRM2, SCD, SKP2 |
| miR-146a-5p | Inhibited | -2.745 | 0.0237 | CCL2, CCNA2, CXCL3, IL6,  KIF22, LBP, PA2G4, PRR15 |
| miR-29b-3p | Inhibited | -2.646 | 0.0374 | ADAMTS15, BCL2L11, Dst, GAS7,  NID1, PLEC, PTEN |
| miR-16-5p | Inhibited | -2.606 | 0.016 | CACNA2D1, ECHDC1,  H3-3A/H3-3B, IGF2R, IL6, NAA15, NPR3, PSAT1, RNASEL, RTN4, SLC12A2, TPM3, TXN2, UCP2 |
| miR-155-5p | Inhibited | -2.447 | 0.0152 | BACH1, BCL2L11, Ccl2, CXCL2,  CXCL3, CYP51A1, GNA13, IL6,  MAFB, MARC1, RHOA, TRIP13 |
| miR-30 | Inhibited | -2.342 | 0.00396 | BCL2L11, Ccl2, IL6, MYH7,  SKP2, TNRC6A |
| miR-26 | Inhibited | -2.205 | 0.00137 | CXCL3, FGF9, IL6, MAP3K2,  PTEN |
| miR-181 | Inhibited | -2.163 | 0.00124 | BCL2L11, E2F5, IL6, KLF6,  MME, MMP14, PTEN, VCAM1 |
| miR-24-3p | Inhibited | -2 | 0.0128 | CCNA2, CDK1, FEN1, IL6 |
| miR-335-3p | Inhibited | -2 | 0.0166 | ADAMTS15, Dst, NID1, PLEC |
| miR-338-3p | Inhibited | -2 | 0.0317 | ADAMTS15, Dst, NID1, PLEC |
| miR-217-5p | Activated | 2 | 0.0000996 | ACACA, FASN, PPARA, SCD |
| miR-873 | Activated | 2 | 0.000174 | Ccl2, CCL2, CXCL3, IL6 |
| miR-223 | Activated | 2.335 | 0.000942 | ALCAM, APOBEC1, CA4, CD274,  GPR65, IL1RN, IL6, LY96, MLKL,  SLC2A4, SOAT1, Tlr13, TLR7 |

**Supplemental Figure 6:** **IPA *in silico* analysis of aorta transcriptomic data showing miRNA networks predicted to be activated or inhibited by CLA supplementation.**


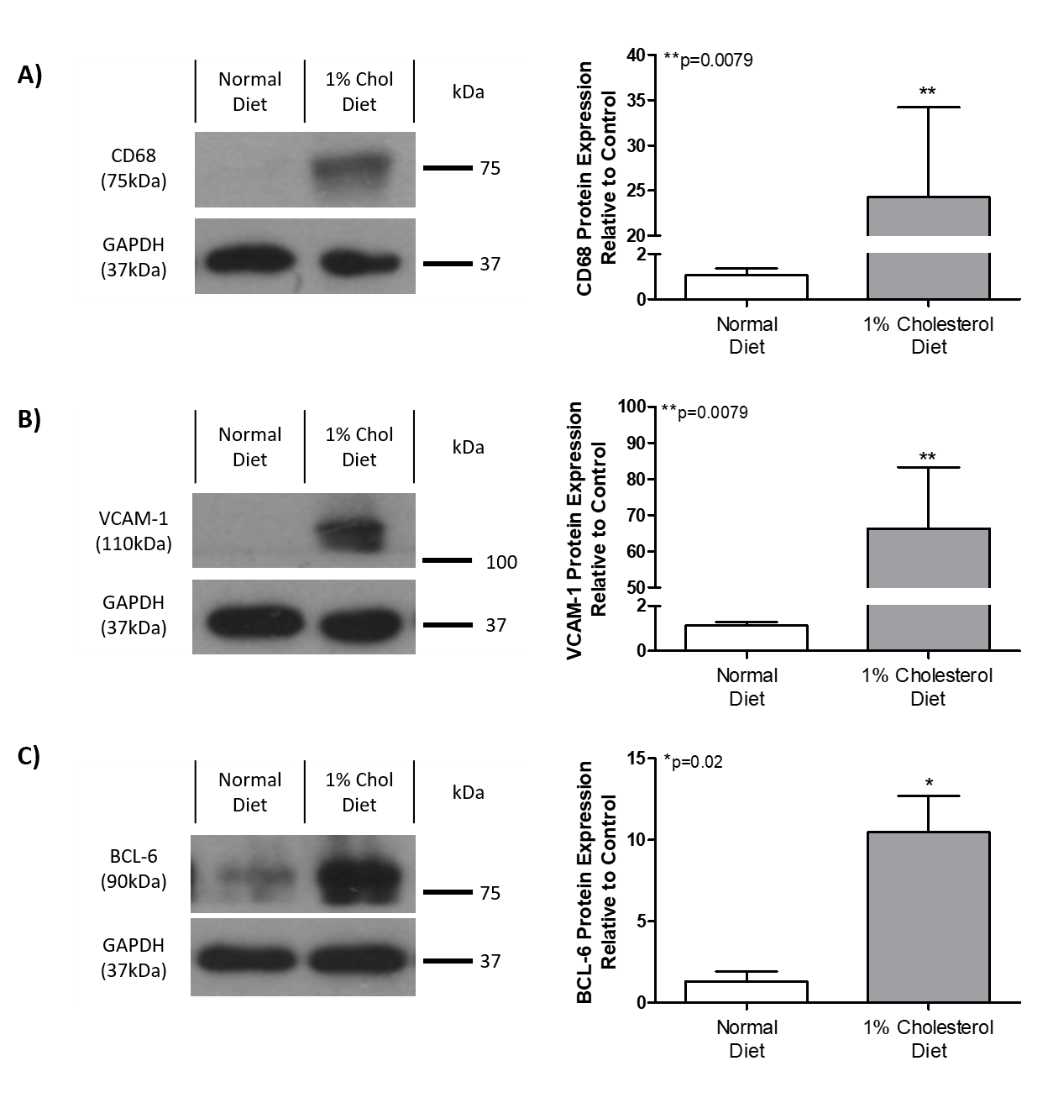


**Supplemental Figure 7: Effect of a 12-week 1% CD on CD68, VCAM-1 and BCL-6 in the aortae of ApoE^-/-^ mice. (A)** CD68, **(B)** VCAM-1 and **(C)** BCL-6 proteins in aortae from mice fed a 12wk normal chow diet or 1% cholesterol diet were analyzed by Western blot. Densitometry analysis was performed, and error bars are representative of 5 mice per group. Statistical analysis was carried out using a nonparametric Mann-Whitney U tests where *p<0.05 and **p<0.01 represent statistical significance in comparison to the normal diet control group.

**
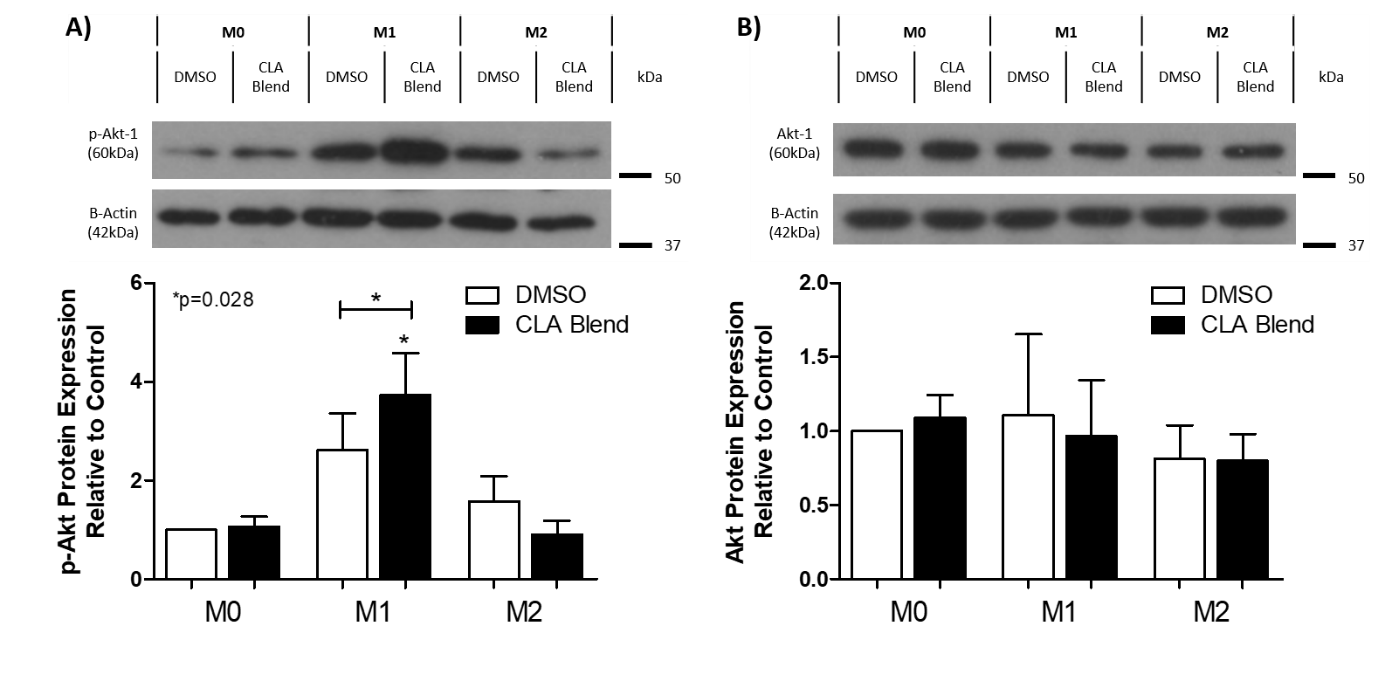
**

**Supplemental Figure 8:** **CLA increases protein expression of p-Akt in polarized M1 THP-1 macrophages.** THP-1 monocytes were polarized into THP-1 macrophages as described previously. Supernatants were removed, and cells were treated with CLA blend (25µmol/L) for 18hr and 0.1% DMSO was used as the control. **(A)** p-Akt (n=5) and **(B)** Akt (n=3) were analyzed by Western blot and densitometry. Statistical analysis was carried out using multiple paired t-tests where *p<0.05 represent statistical significance. * over the columns represents statistical significance when compared to the control group M0 treated with DMSO. Capped lines indicate comparisons between other groups.


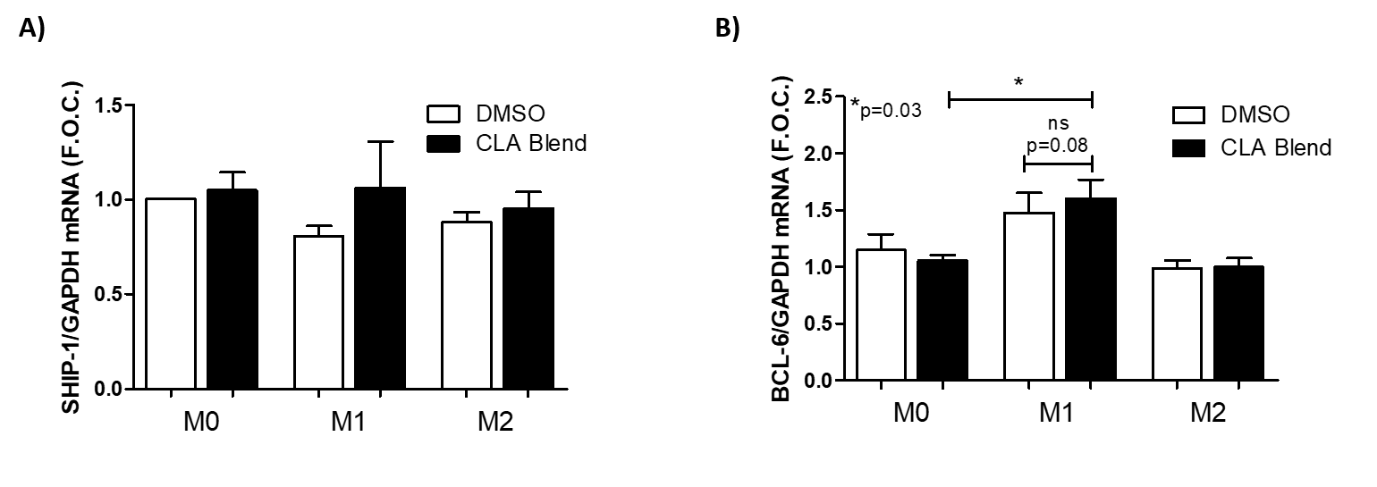


**Supplemental Figure 9:** **CLA effects on mRNA expression of miR-155 targets, BCL-6 and SHIP-1, in polarized THP-1 macrophages.** Polarized THP-1 macrophages were treated with CLA blend (25µmol/L) for 18hr using DMSO (0.1%) as the control. mRNA was analyzed by qRT-PCR for **(A)** SHIP-1 **(B)** BCL-6 (n=5). Error bars are representative of four independent experiments carried out in duplicate and averaged and graphed as fold over control (F.O.C.). Statistical analysis was carried out using multiple paired t-tests where *p<0.05 represent statistical significance. * over the columns represents statistical significance when compared to the control group M0 treated with DMSO. Significance between other groups is indicated by capped lines.

**
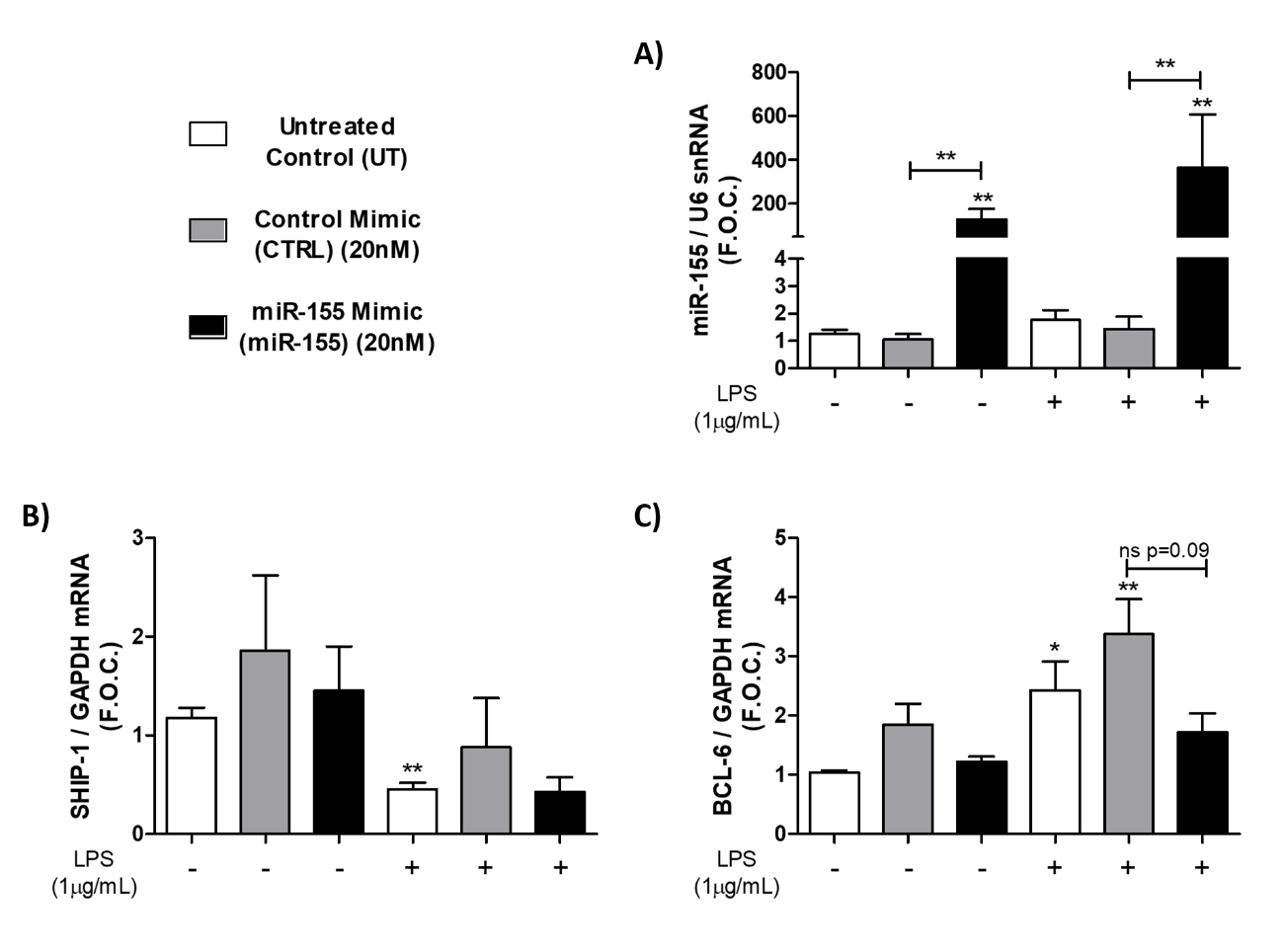
**

**Supplemental Figure 10: Transfection of miR-155 mimic and treatment with LPS altered SHIP-1 and BCL-6 in human atherosclerotic plaques.** As described in supplemental methods 1, carotid plaque specimens were dissected and transfected with 20nmol/L of miR-155 mimic for 18hr in serum-free RPMI in duplicate. Scrambled negative control mimic and Lipofectamine^TM^ 2000 only (untreated control) were used as the controls. Plaques were then treated with 1μg/mL of LPS for 6hr. Plaques were homogenized and RNA was isolated *via* TRIzol^TM^ extraction. Transfection efficiency of **(A)** miR-155 mimic (n=5), **(B)** BCL-6 (n=5) and **(C)** SHIP-1 (n=5) were analyzed by qRT-PCR where duplicates were averaged and graphed as fold over control (F.O.C.). Statistical analysis was performed using multiple nonparametric Mann-Whitney tests where *p<0.05 and **p<0.01 represent statistical significance. * over the columns represent statistical significance when compared to the untreated control group without LPS. Statistical significance between groups is indicated by capped lines.

**
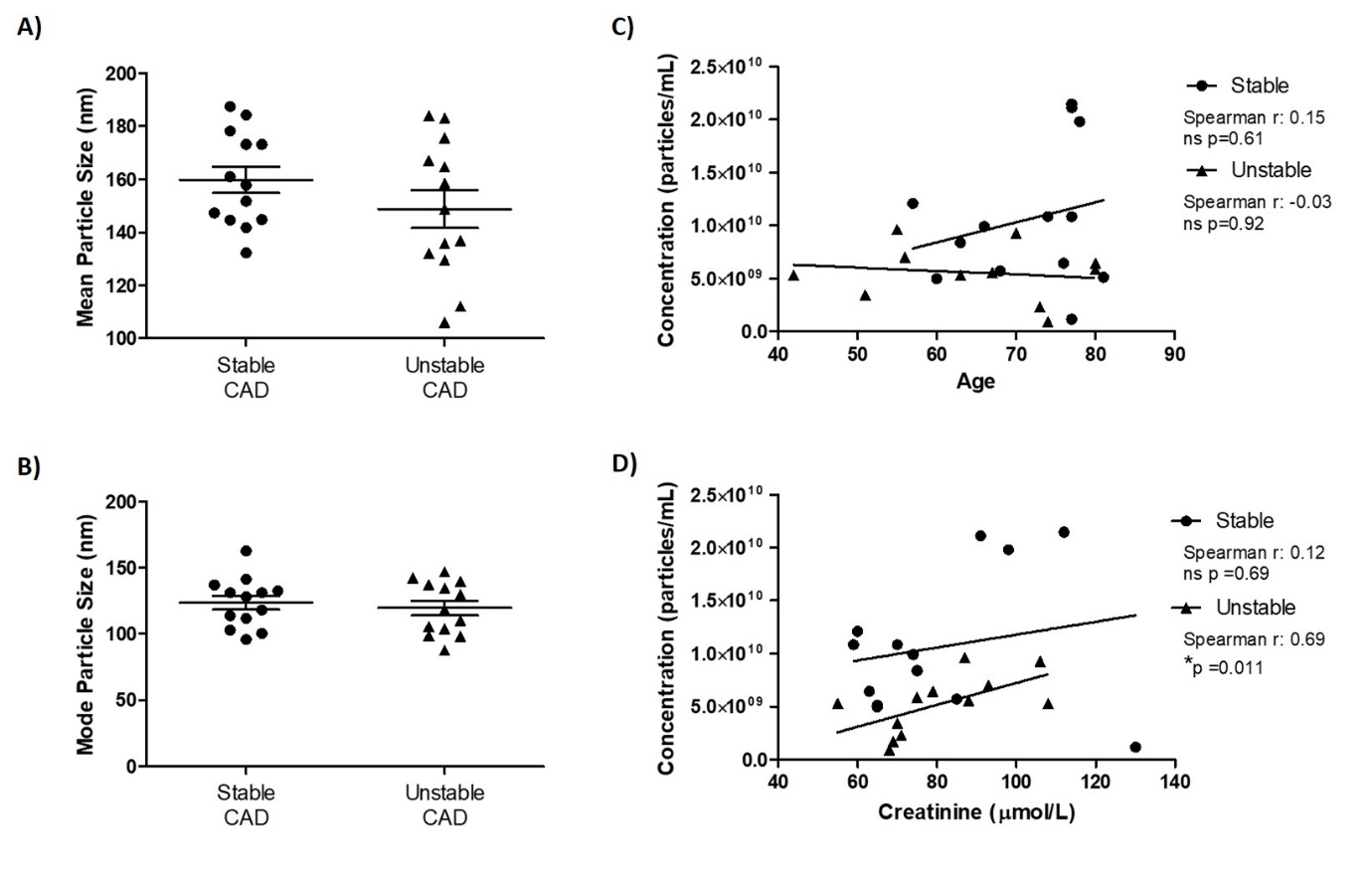
**

**Supplemental Figure 11: Comparison of CAD patient uEV size and uEV concentration with creatinine concentration and age as analyzed by NTA.** The **(A)** mean and **(B)** mode uEV particle size of the stable (n=13) and unstable (n=12) cohorts. **(C)** Correlation analysis of age in both cohorts of patients with total particle concentration. **(D)** Correlation analysis of creatinine concentration in both cohorts of patients with total particle concentration. Linear regression analysis was performed to assess correlations between the age and creatinine concentration and the particle concentration using GraphPad Prism 5.0c. Statistical analysis was performed using a non-parametric Mann-Whitney U test (A, B) and a Spearman test (C, D) where *p<0.05 represents statistical significance in comparison to the respective controls. A statistically non-significant change is represented by ns.


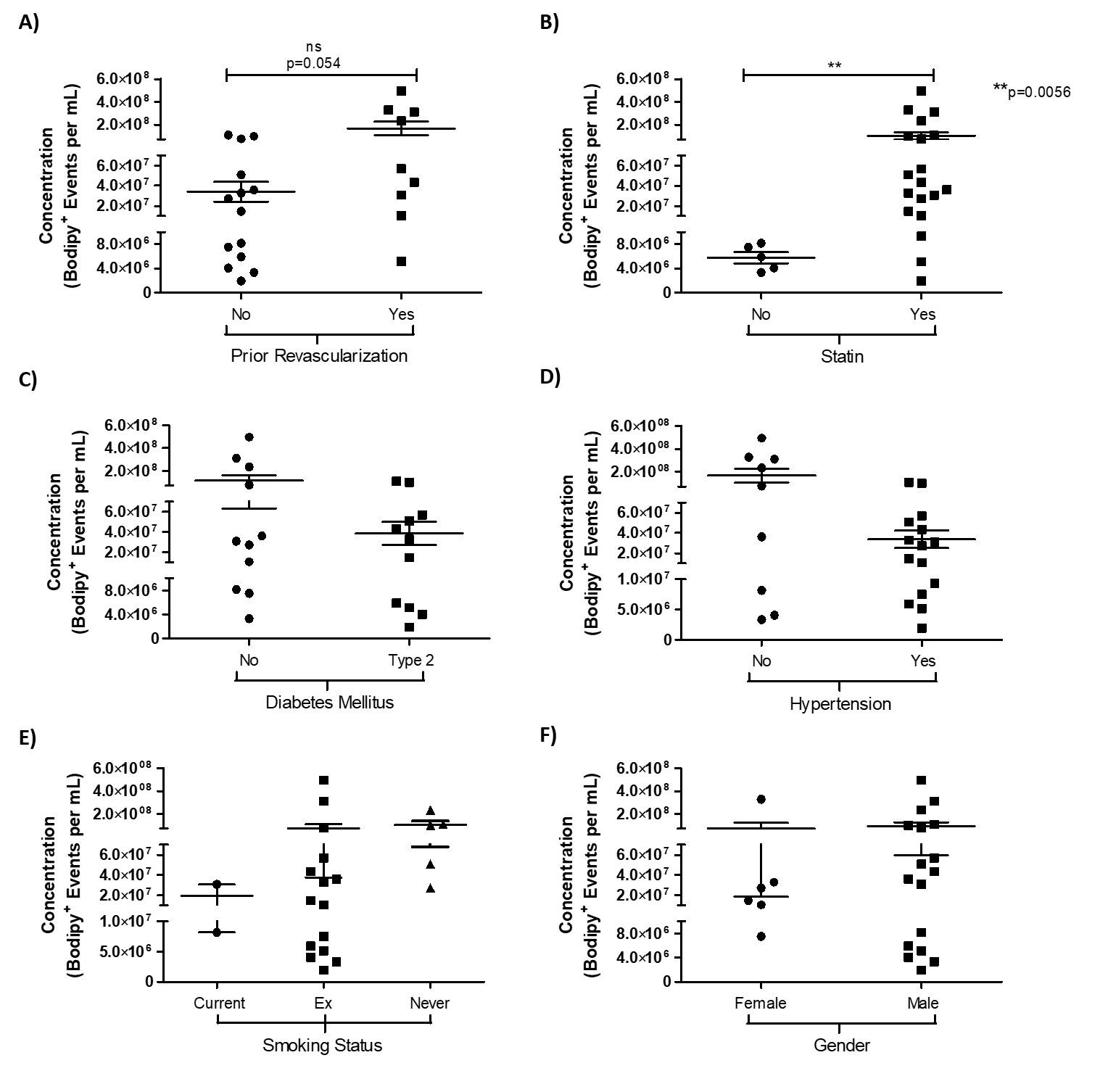


**Supplemental Figure 12: Analysis of** **the association between uEV concentration and multiple patient clinical parameters.** The concentration of uEVs as determined by flow cytometry was analyzed in comparison to multiple patient parameters as outlined in Table 1**.** Bodipy^+^ uEV concentration from each patient was graphed based on their clinical parameters; **(A)** having previously underwent prior revascularization surgery, **(B)** currently prescribed statin medication, **(C)** diagnosed with diabetes mellitus, **(D)** diagnosed with hypertension, **(E)** based on their smoking status (where Ex means the patient has stopped smoking), **(F)** based on their gender. Statistical analysis was performed using a nonparametric Mann-Whitney U test where *p<0.05 and **p<0.01 represent statistical significance in comparison to the respective controls. Statistically non-significant change is represented by ns. capped lines indicate comparisons between groups.

**
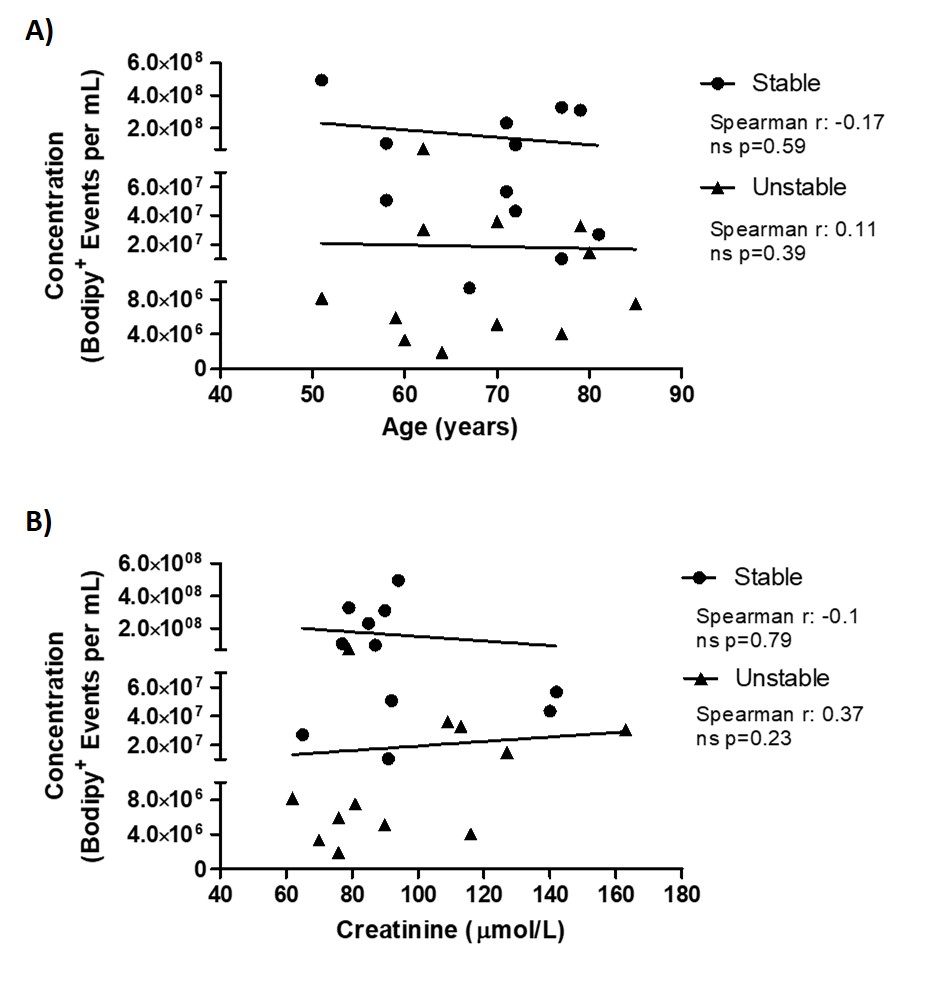
**

**Supplemental Figure 13: Comparison of CAD patient uEV concentration as analyzed by flow cytometry with creatinine concentration and age. (A)** Correlation analysis of both stable (n=12) and unstable(n=12) cohorts comparing age with Bodipy^+^ uEV concentration. **(B)** Correlation analysis of both stable(n=12) and unstable(n=12) patients comparing creatinine concentration with concentration of Bodipy^+^ uEV events per mL. Linear regression analysis was performed to assess correlations between the patient demographics and the Bodipy^+^ uEV concentration using GraphPad Prism 5.0c. Statistical analysis was performed using a Spearman test where *p<0.05 represent statistical. A statistically non-significant change is represented by ns.
